# Mps1^Mph1^ Kinase Phosphorylates Mad3 to Inhibit Cdc20^Slp1^-APC/C and Maintain Spindle Checkpoint Arrests

**DOI:** 10.1371/journal.pgen.1005834

**Published:** 2016-02-16

**Authors:** Judith Zich, Karen May, Konstantinos Paraskevopoulos, Onur Sen, Heather M. Syred, Sjaak van der Sar, Hitesh Patel, James J. Moresco, Ali Sarkeshik, John R. Yates, Juri Rappsilber, Kevin G. Hardwick

**Affiliations:** 1 Wellcome Trust Centre for Cell Biology, Institute of Cell Biology, University of Edinburgh, Edinburgh, United Kingdom; 2 Edinburgh Cancer Research UK Centre, Institute of Genetics and Molecular Medicine, University of Edinburgh, Edinburgh, United Kingdom; 3 Scripps Research Institute, La Jolla, California, United States of America; 4 Department of Bioanalytics, Institute of Biotechnology, Technische Universitat Berlin, Berlin, Germany; University of California San Francisco, UNITED STATES

## Abstract

The spindle checkpoint is a mitotic surveillance system which ensures equal segregation of sister chromatids. It delays anaphase onset by inhibiting the action of the E3 ubiquitin ligase known as the anaphase promoting complex or cyclosome (APC/C). Mad3/BubR1 is a key component of the mitotic checkpoint complex (MCC) which binds and inhibits the APC/C early in mitosis. Mps1^Mph1^ kinase is critical for checkpoint signalling and MCC-APC/C inhibition, yet few substrates have been identified. Here we identify Mad3 as a substrate of fission yeast Mps1^Mph1^ kinase. We map and mutate phosphorylation sites in Mad3, producing mutants that are targeted to kinetochores and assembled into MCC, yet display reduced APC/C binding and are unable to maintain checkpoint arrests. We show biochemically that Mad3 phospho-mimics are potent APC/C inhibitors *in vitro*, demonstrating that Mad3p modification can directly influence Cdc20^Slp1^-APC/C activity. This genetic dissection of APC/C inhibition demonstrates that Mps1^Mph1^ kinase-dependent modifications of Mad3 and Mad2 act in a concerted manner to maintain spindle checkpoint arrests.

## Author Summary

When cells divide they need to ensure that a complete copy of their genetic material is transmitted to both daughter cells. Cells have evolved many controls to ensure that every division is carried out with very high fidelity. The spindle checkpoint is one such control, which acts as a surveillance system during mitosis. Defects in this checkpoint control lead to unequal segregation of DNA/chromosomes, termed aneuploidy, which is responsible for human birth defects and is very common in tumour cells.

The molecular components of the spindle checkpoint, identified initially through yeast genetics, include several protein kinases. Surprisingly few of their substrates have been identified. Here we identify the checkpoint protein Mad3 as an important substrate of the Mps1^Mph1^ kinase. We show that Mps1^Mph1^-dependent modification of Mad3 and Mad2 acts to delay cell division in situations where the genetic material would not be equally inherited by daughter cells. This delay enables the cell to correct any problems within the division machinery and thus avoid aneuploidy.

## Introduction

Defects in chromosome segregation result in aneuploidy, which can lead to disease or cell death [1,2,3]. Mitosis is an extremely complicated and well orchestrated stage of the cell cycle, and many controls are employed to ensure its high fidelity. One of the major controls is the spindle checkpoint which acts as a surveillance system monitoring kinetochore-microtubule attachments. It delays anaphase onset until all sister-chromatid pairs are bi-oriented on the mitotic spindle [4,5,6]. Anaphase onset is initiated by an E3 ubiquitin ligase, known as the anaphase promoting complex or cyclosome (APC/C), and its activating co-factor Cdc20^Slp1^ [7,8]. Cdc20^Slp1^-APC/C targets the separase inhibitor securin and the CDK1 activating subunit cyclin B for destruction by the 26S proteasome [9,10,11]. Once securin is destroyed, cohesin is cleaved and sister chromatids separate and segregate in anaphase [12].

The spindle checkpoint utilises several mechanisms to efficiently inhibit Cdc20^Slp1^-APC/C. These include sequestration and phosphorylation of Cdc20^Slp1^ [13], formation of the mitotic checkpoint complex (MCC) that typically consists of the spindle checkpoint proteins Mad2, BubR1/Mad3, Bub3 and Cdc20^Slp1^ [14,15,16,17,18] and MCC binding to the APC/C [19,20,21,22]. Spindle checkpoint components are highly conserved from yeast to humans, and include the *MAD* (mitotic-arrest deficient) and the *BUB* (budding uninhibitied by benzimidazole) genes [23,24]. Spindle checkpoint kinases include Mps1^Mph1^, Bub1 and Aurora B^Ark1^, but their precise signalling roles remain far from clear [25,26,27,28].

*S*.*pombe* Mph1 is a structural and functional homologue of *S*.*cerevisiae* Mps1, but it is neither required for spindle pole duplication nor essential for cell viability [29]. Homologues in higher organisms have been shown to be essential for the spindle checkpoint and for efficient chromosome segregation [30,31,32,33,34,35,36].

The fission yeast Mps1^Mph1^ substrates identified to date are KNL1^Spc7^ [37,38] and Mad2 [39]. KNL1^Spc7^ is an important Mps1^Mph1^ substrate at kinetochores, which when phosphorylated becomes the kinetochore binding site for the Bub1-Bub3 complex [37,38]. This role is conserved in budding yeast and vertebrates [38,40,41,42], and structural studies have shown that it is Bub3 that binds directly to the MELT motifs after they are phosphorylated by Mps1^Mph1^ [43,44]. In budding yeast it has been shown that Mps1^Mph1^ kinase then phosphorylates kinetochore-bound Bub1 to enhance the recruitment of the Mad1-Mad2 complex [45], but this remains to be confirmed in other systems. Thus Mps1^Mph1^ kinase has a key role in assembling the checkpoint signalling scaffold (KNL1^Spc7^-Bub1-Mad1) at yeast kinetochores. Additional substrates of Mps1^Mph1^ kinase have been identified, including spindle pole body components [46,47], the Borealin component of the human chromosomal passenger complex (CPC) [30], and the Dam1 [48] and Ndc80 [49] kinetochore proteins. Thus it is clear that Mps1^Mph1^ kinase is a central player in mitotic regulation [27].

In a previous study we identified Mad2 as an Mps1^Mph1^ checkpoint substrate and described the *mad2-S92A* allele that displayed reduced MCC-APC/C binding and reduced ability to maintain spindle checkpoint arrest [39]. Here we demonstrate that Mad3 is another important checkpoint substrate for Mps1^Mph1^ kinase. Twelve *in vivo* phosphorylation sites were mapped in Mad3, probably due to the action of multiple protein kinases (CDK, Mph1 and Ark1) and sixteen phospho-modifications were generated and mapped *in vitro* through the direct action of Mps1^Mph1^ kinase. A series of phosphorylation site mutants were generated, and mutations in the C-terminus of Mad3 were found to have impaired checkpoint function. These defects were compounded in strains where the *mad3-C9A* allele was combined with *mad2-S92A*. Importantly, when C-terminal Mad3 phosphomimics were directly tested *in vitro* they were found to be potent APC/C inhibitors. We propose that Mps1^Mph1^ kinase phosphorylates multiple components of the fission yeast MCC to stabilise its interaction with the APC/C and thereby maintain spindle checkpoint arrests.

## Results

### Mad3p is phosphorylated by Mps1^Mph1^ kinase

We previously reported that Mad2p is phosphorylated by Mps1^Mph1^ kinase, and that mutation of Mad2p phosphorylation sites partially abrogated the spindle checkpoint [39]. However, the checkpoint phenotype of Mps1^Mph1^ kinase-dead alleles was much stronger, indicating that other relevant Mps1^Mph1^ substrates remain to be found. Whilst phosphorylation of KNL1^Spc7^ at kinetochores may account for some of this checkpoint function [37,38], there was still a defect apparent in the *mps1∆ spc7-T12E* strain where all the Mps1^Mph1^ sites in KNL1^Spc7^ had been mutated to phosphomimic (Glutamate) residues [38], again arguing for additional Mps1^Mph1^ substrates. The *bub3∆* phenotype, where Bub1p, Mad3p, Mad1p and Mad2p all fail to be recruited to kinetochores yet the checkpoint arrest remains robust, also argues against an absolute requirement for checkpoint proteins to be recruited to KNL1^Spc7^ and kinetochores in fission yeast [50,51,52]. In the absence of Mps1^Mph1^ kinase activity the mitotic checkpoint complex (MCC) is not tightly associated with APC/C [39], so we tested whether fission yeast Mad3p is also a substrate of Mps1^Mph1^ kinase.

**Fig 1:**
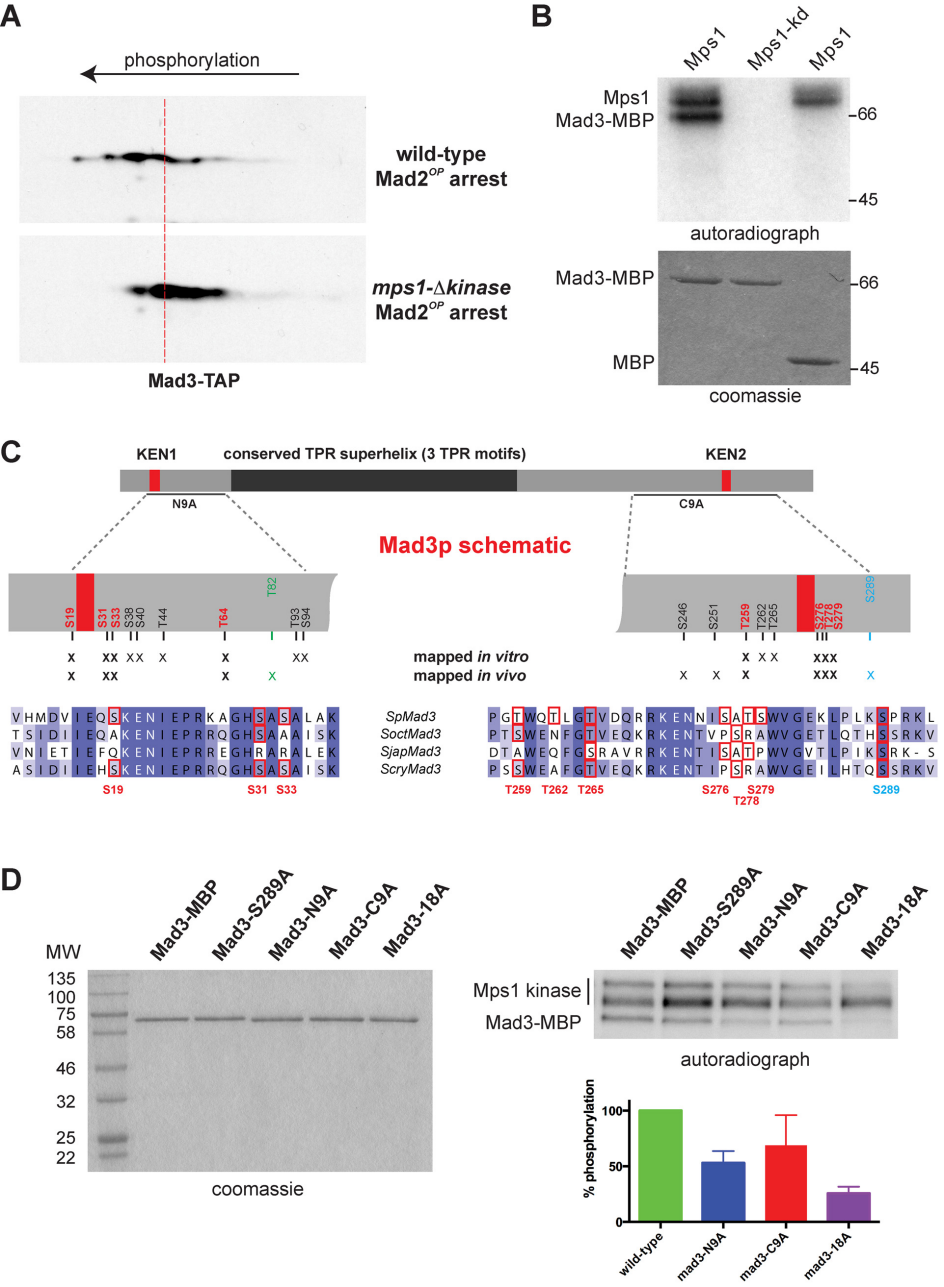
Mad3p phosphorylation. A. Mad3p modification is Mps1^Mph1^-dependent. Wild-type and *mph1-Δkinase* strains expressing Mad3-TAP were arrested in mitosis by overproducing Mad2p (Mad2^OP^) from pREP1-Mad2. Yeast extracts were made, separated by 2D-PAGE and immunoblotted with peroxidase-conjugated anti-peroxidase antibody to detect Mad3-TAP isoforms. B. Mps1^Mph1^ kinase phosphorylates Mad3p *in vitro*. Mps1^Mph1^-SZZ and Mps1^Mph1^kinase-dead-SZZ were purified from yeast extracts then used to phosphorylate Mad3-MBP or MBP. C. Mad3p phosphorylation sites. Mad3p schematic, highlighting KEN boxes, the TPR region, and positions of the N9A and C9A alleles. Mass spectrometry identified many sites in Mad3p: those sites identified both in the *in vitro* Mps1^Mph1^ kinase assay and *in vivo* are highlighted in red. The 16^th^ *in vitro* Mps1^Mph1^ site (T195) is not labelled on this schematic. There is a putative Ark1 site in green (T82) and CDK site (S289) in blue. Alignments of Mad3p KEN box regions from the four sequenced fission yeasts (*Sp Schizosaccharomyces pombe*, *Soct Schizosaccharomyces octosporus*, *Sjap Schizosaccharomyces japonicus*, *Scry Schizosaccharomyces cryophilus–*sequences available from the Broad Institute, USA) highlighting clusters of phosphorylation sites flanking the KEN boxes. D. Mps1^Mph1^ kinase phosphorylates Mad3p alanine mutants at reduced levels *in vitro*. The indicated recombinant Mad3-MBP fusion proteins were purified from bacteria and used as substrates in an Mps1^Mph1^-SZZ kinase assay. The kinase assay was performed 4 times, a representative coomassie gel and autoradiograph are shown here, and their quantitation plotted (mean with SEM).

First we analysed the *in vivo* dependence of Mad3p modification on Mps1^Mph1^ kinase. No clear gel shifts were apparent for Mad3p on regular SDS-PAGE and so we employed 2D gel-immunoblotting, comparing Mad3p modification in wild-type cells and cells lacking Mps1^Mph1^ kinase activity. As *mps1-kd* cells are unable to checkpoint arrest [39] we compared Mad3p modification after cells had been mitotically arrested through overexpression of Mad2p [53]. Fig 1A shows a clear charge-related shift for Mad3p isoforms in the two mitotic yeast extracts, demonstrating that Mad3p is modified in an Mps1^Mph1^ -dependent manner in fission yeast during mitosis. Next we carried out *in vitro* Mps1^Mph1^ kinase assays using recombinant Mad3-MBP as substrate (Fig 1B). These assays were analysed by mass-spectrometry, both with and without phosphopeptide enrichment on titanium oxide beads (see Materials and Methods). Under these conditions, sixteen *in vitro* Mps1^Mph1^ sites in Mad3p (see Fig 1C and S2 Fig for spectra) were identified.

To confirm these are phosphorylation sites *in vivo* we purified checkpoint complexes (purifying Mad3-TAP and Apc4-TAP) containing Mad3p from both cycling and checkpoint arrested (*nda3*) fission yeast cells, and analysed them by MudPIT [54]. We were able to confirm that eight of the *in vitro* Mps1^Mph1^ sites (S19,S31,S33,T64,T259,S276,T278,S279) are also modified in Mad3p purified from yeast cells (*in vivo)*. In addition, four other *in vivo* sites (T82, S246, S251 and S289) were found in Mad3p, identifying modifications that are presumably made by other protein kinases. Whilst we do not know the identity of the other Mad3p kinase(s), we think it extremely likely that S289 is a CDK site and note that T82 fits the Aurora (Ark1) consensus. Mutation of the CDK site alone had no detectable phenotype (see S3 Fig). Several of the Mps1^Mph1^ sites are conserved in fission yeasts (see S1 Fig). Whilst none of the Mps1^Mph1^ sites are particularly well conserved in other organisms, we think it noteworthy that there are conserved clusters of Mps1^Mph1^ phosphorylatable residues flanking both of the Mad3p KEN boxes and that seven of these clustered residues (S19,S31,S33,T259,S276,T278,S279) were only modified in mitotic samples. The Mad3p phosphorylation sites that we identified are summarised in Fig 1C and S1B Fig.

### Mad3p phosphorylation site mutants are checkpoint defective

To test the physiological relevance of these Mps1^Mph1^ dependent modifications, we constructed mutations in Mad3p phosphorylation sites in various combinations, substituting serine and threonine residues with non-phosphorylatable alanines (*mad3-N9A*, *mad3-C9A* and *mad3-18A –*see S1A Fig for full fission yeast Mad3p alignments). *In vitro* kinase assays were performed using Mps1^Mph1^ kinase and recombinant Mad3-MBP fusion proteins containing these mutations (Fig 1D). Quantitation demonstrated that the N-terminal sites account for ~50% of the *in vitro* modification and the C-terminal sites ~30%. Importantly, mutation of all 18 sites reduced phosphorylation by ~80%, indicating that we have identified almost all of the *in vitro* sites for Mps1^Mph1^ kinase.

**Fig 2:**
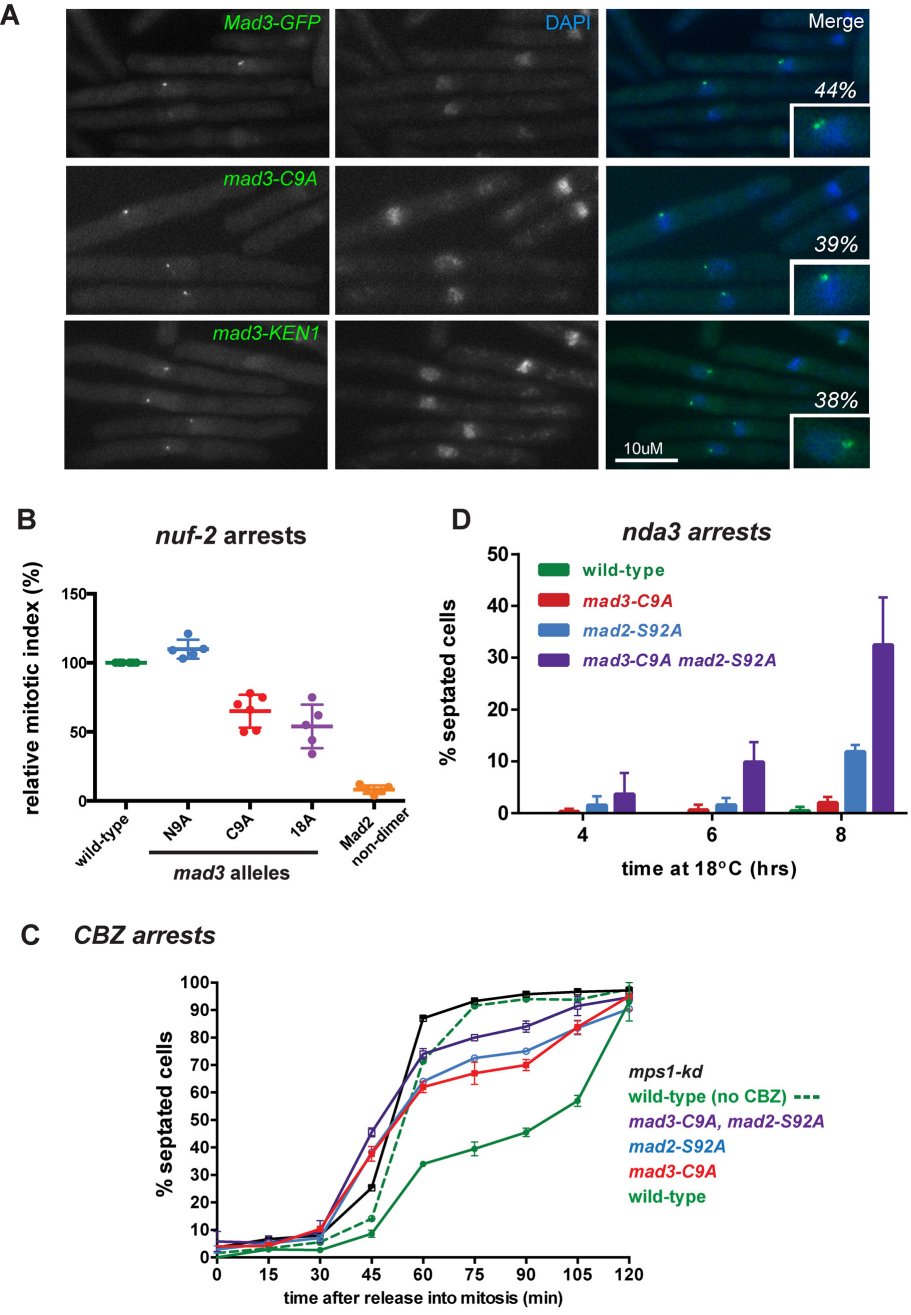
*mad3* phospho-mutants are localised and assembled into MCC complexes effectively, but *mad2 mad3* double phospho-mutants have severe defects in the maintenance of checkpoint arrests. A. Mutant Mad3 proteins are efficiently targeted to kinetochores. GFP fusions were constructed for all three phospho-mutants and -KEN mutants. *cdc25* strains were synchronised in G2 and then released into mitosis for GFP imaging. Images shown were taken 20 mins after release. DNA was stained with DAPI. The inset images are labelled with the % of cells having Mad3-GFP kinetochore foci. B. *nuf2-3* strains containing *mad3-N9A*, *mad3-C9A* and *mad3-18A* alleles or the *mad2* non-dimerising mutant (M2 dimer) were shifted to 36°C for 4 hrs. Cells were then fixed and microtubules were stained with anti-tubulin antibodies. The relative mitotic index (cells containing short metaphase spindles) is plotted after normalising the % of mitotic spindles in the *nuf2-3* control strain (which has a functional checkpoint) to 100. >100 cells were scored for each sample and this experiment repeated 5 times (scatter dot plot: lines at mean with SD). C. *cdc25* strains containing *mad3-C9A*, *mad2-S92A*, *mad3-C9A* and *mad2-S92A*, or *mps1*^*mph1*^*-kd* mutations were synchronised in G2, released in mitosis and then challenged to arrest in response to the anti-microtubule drug carbendazim (CBZ). Failure to maintain mitotic checkpoint arrest leads to septation and this was scored at 15 min time-intervals, by fixing cells and staining with calcofluor (see supp. data for images). This experiment was repeated twice and >100 cells scored for each strain at each time point (plotted as mean with SEM). D. *nda3* strains containing *mad3-C9A*, *mad2-S92A*, or *mad3-C9A* and *mad2-S92A* mutations were grown to log phase and then shifted to 18°C to analyse their ability to metaphase-arrest for several hours in response to the absence of microtubules. Failure to maintain arrest results in septation. This experiment was repeated twice and 100 cells scored for each strain at each time point (plotted as mean with SEM).

These mutations were then tested in yeast, by replacing the endogenous *mad3+* gene with the mutant alleles. They had no effect on Mad3 protein targeting to kinetochores (Fig 2A) nor lead to significant growth defects when grown on plates containing the anti-microtubule drug benomyl (see S3 Fig). However, when we employed more sensitive assays for loss of spindle checkpoint function significant defects became apparent. The *mad3* alleles were tested for their ability to checkpoint arrest cells containing kinetochore defects, by employing the temperature-sensitive *nuf2-3* allele [55] at 32°C, where typically ~30% of cells should arrest with short spindles after 4 hours (Fig 2B). This experiment demonstrates that whilst mutation of nine N-terminal phosphorylation sites in Mad3p had no effect on the ability of cells to arrest in response to kinetochore attachment defects, mutation of nine C-terminal sites reduced the efficiency of the *nuf2-3* arrest by ~40%. Mutating all eighteen sites to alanine made the arrest even worse (~50% efficiency), but this further decrease is hard to interpret as it could partly be due to the reduced stability of this mutant Mad3 protein. The levels of the mutant Mad3 proteins were ~75% for *mad3-C9A* but only ~20% for *mad3-18A*, and others have shown that when Mad3p falls below 30% of its normal level it can perturb the checkpoint [56]. We propose that phosphorylation of Mad3p, in particular towards its C-terminus, can enhance its ability to maintain a checkpoint arrest and thereby delay anaphase onset. These results lead us to focus on the C-terminal Mad3p phosphorylation sites for the rest of this study.

To confirm their checkpoint defect we challenged mitotic *mad3-C9A* cells growing in liquid culture with the anti-microtubule drug carbendazim (CBZ) and analysed their rate of septation (mitotic exit). Failure to maintain spindle checkpoint arrest leads to an increase in septation index. We pre-synchronised *cdc25* strains in G2 (growing them for 3.5 hrs at 36°C) and then released them (shifting down to 24°C) to undergo synchronous mitoses. Twenty minutes after G2 release we added 75μg/ml CBZ to depolymerise their microtubules. Using this assay we compared the *mad3-C9A* allele with the *mad2-S92A* and *mps1-kd* alleles we have previously described [39]. Fig 2C shows that the *mad3-C9A* cells fail to effectively maintain a checkpoint arrest and septate with very similar kinetics to *mad2-S92A*. We made a double mutant (*mad3-C9A mad2-S92A*) and found that these cells have a synthetic checkpoint defect and septate at a faster rate, in between that of the single mutants and the *mps1-kd* allele (see S4A Fig for representative images). We note that in some of these septating cells Mad3-GFP can still be detected on kinetochores after cells have septated (see S4B Fig): this observation is consistent with such cells having a downstream defect in *maintenance* of their checkpoint arrest, rather than a Mad3 defect in checkpoint signalling at kinetochores and/or their checkpoint being satisfied due to proper kinetochore-microtubule attachments being formed.

The synthetic effect of the double mutant was also observed when the checkpoint was activated in the *nda3* mutant, with 33% of *mad3-C9A mad2-S92A* cells unable to maintain a checkpoint arrest after 8 hours (compared to 0.5% of wild type, 2% of *mad3-C9A* and 11% of *mad2-S92A* leaking through the arrest, Fig 2D). We conclude that reduced phosphorylation of either Mad3p or Mad2p impairs maintenance of a checkpoint arrest, and that reduced phosphorylation of both proteins further reduces their ability to maintain a robust checkpoint response. Our interpretation is that Mad3p and Mad2p are both phosphorylated by Mps1^Mph1^ kinase and that these modifications act in concert to inhibit Cdc20^Slp1^-APC/C.

### Mad3p phosphorylation is important for MCC-APC/C binding

**Fig 3:**
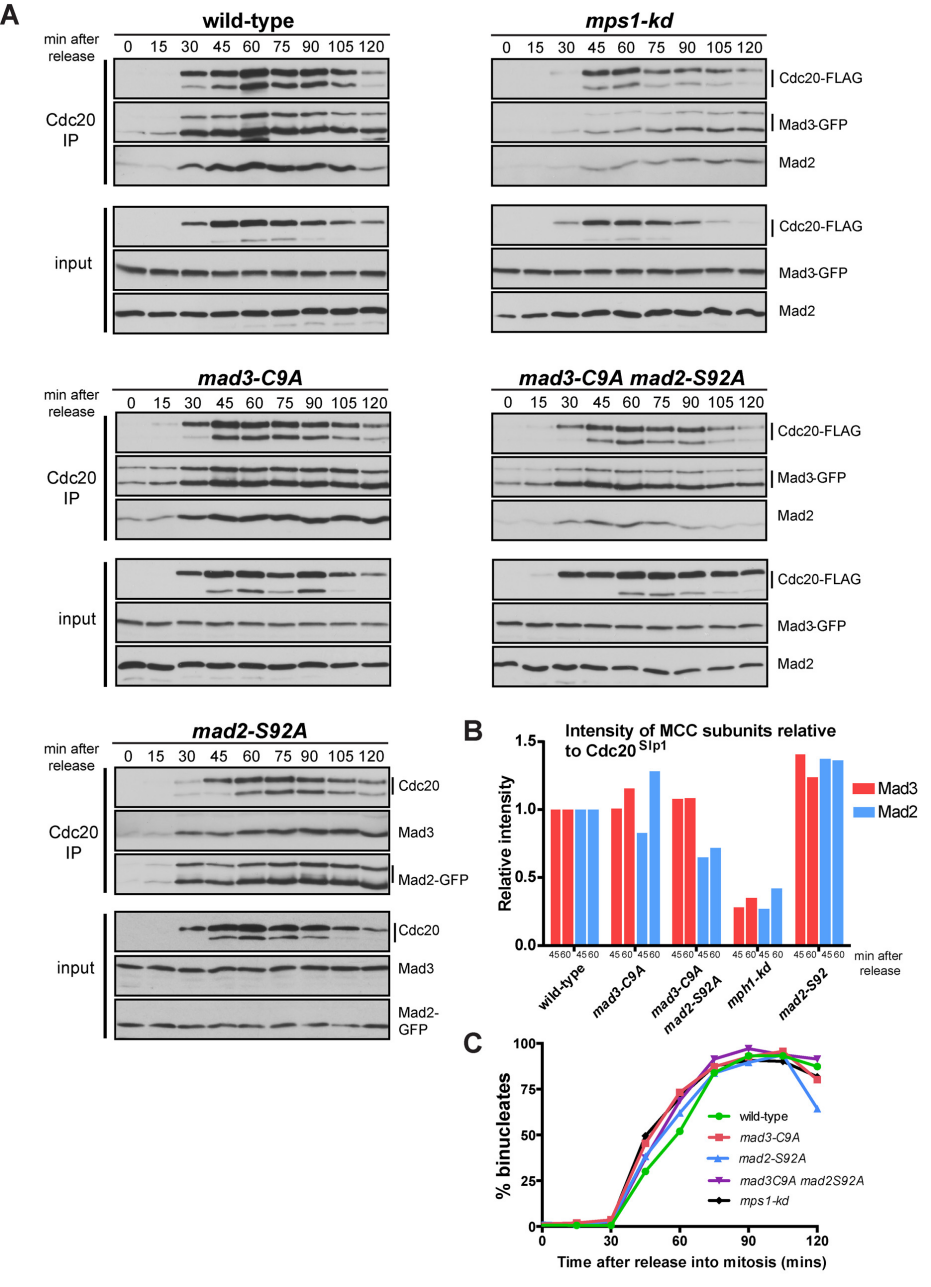
MCC assembly defects are rather minor in the *mad3-C9A* and *mad2 mad3* double phospho-mutants when compared to *mps1-kd*. A. The *cdc25* strains indicated were pre-synchronised in G2 by shifting to 36°C for 3.5 hours. They were then released at 25°C and time points taken every 15 minutes. Native extracts were made and Cdc20^Slp1^ was immunoprecipitated with anti-Flag antibodies. Immunopreciptates were separated by SDS-PAGE and immunoblotted for Cdc20^Slp1^, Mad3p (anti-GFP) and Mad2p. B. These immunoblots were quantitated and plotted as the intensity of Mad3 or Mad2 relative to the Cdc20^Slp1^ level at each time point (45 and 60 minutes after release into mitosis). All values were then compared to wild-type–see Materials and Methods for details. C. Cell cycle progression was scored by DAPI staining methanol fixed cells and quantifying the % of bi-nucleate cells in the population at each time point (200 cells were scored at each time point).

Next we wanted to analyse whether the phosphorylation of the Mad3p C-terminus was relevant to MCC assembly and/or stabilisation. To do this, we carried out *cdc25* (G2) block and release time courses and monitored MCC assembly in the *mad3-C9A*, *mad2-S92A*, *mad3-C9A mad2-S92A* double mutants and *mps1-kd* mutants. Cdc20^Slp1^-pull downs, employing an internally-engineered 3xFLAG tag (see Materials and Methods) demonstrated that MCC was assembled with normal kinetics in *mad3-C9A* and *mad2-S92A* alleles (Fig 3A), but that there were slightly reduced MCC levels in the double mutant. We note that MCC levels dropped significantly, by 70% compared to wild-type at 45 minutes, in the *mps1-kd* strain (Fig 3B). Quantitation of the Cdc20^Slp1^ protein levels in extracts through these time courses (see S6 Fig) shows that they are only reduced in the *mps1-kd* mutant, and there only by 20% which can not explain why the MCC levels drop by 70–80% in this strain. No clear effect on mitotic progression was observed in these unperturbed mitoses (Fig 3C), suggesting that it is not MCC levels that are rate-limiting.

We conclude that phosphorylation of neither the C-terminus of Mad3p nor of Mad2p are critical for MCC assembly. Even when both Mad3p and Mad2p phosphorylation are impaired, only slightly reduced MCC levels result (20–30% reduction in bound Mad2p relative to wild-type levels). This compares with the far more significant reduction in MCC levels observed in the *mps1-kd* cells.

**Fig 4:**
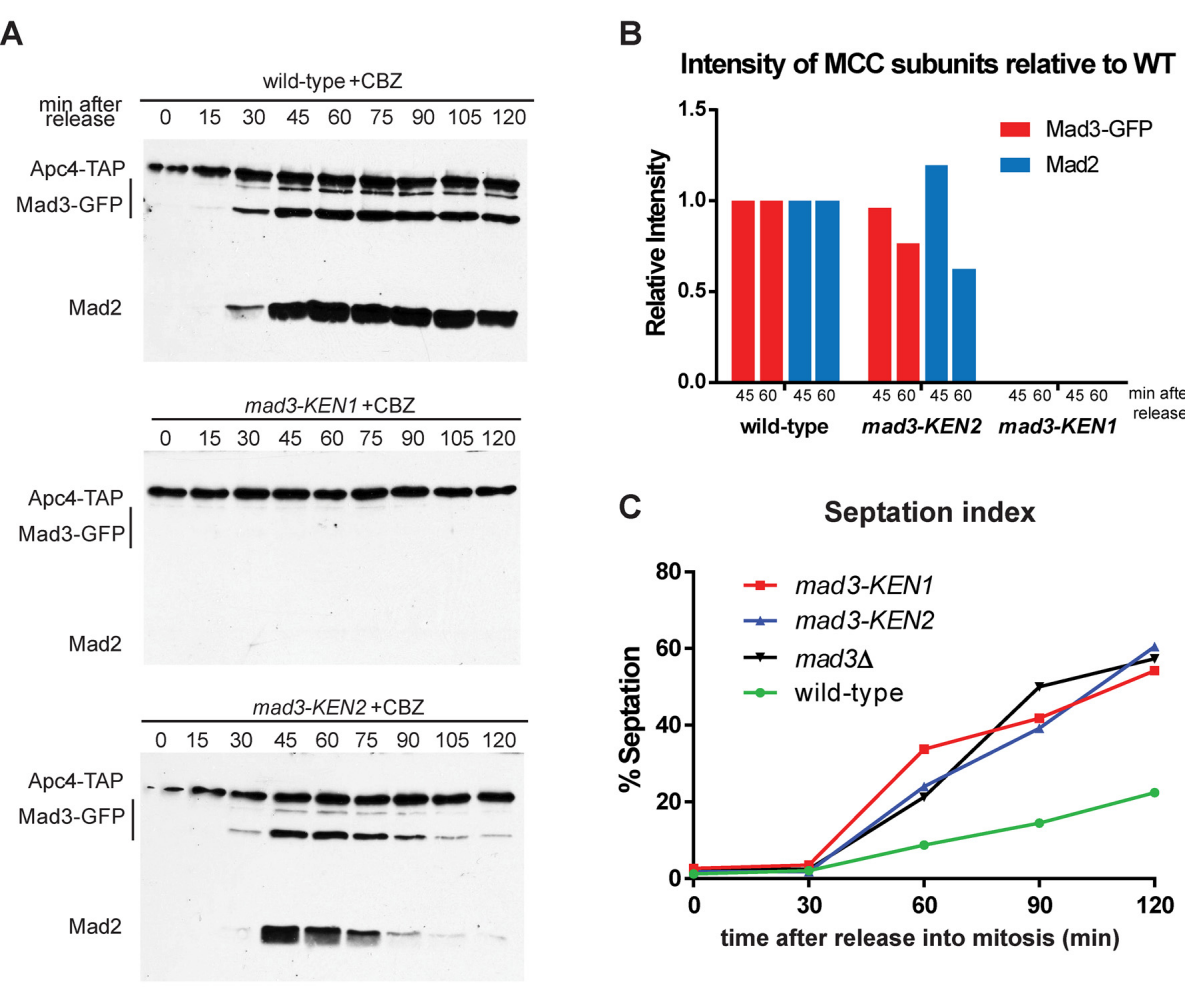
*mad3-KEN* mutants have APC/C binding defects. A. The *cdc25* strains indicated were pre-synchronised in G2 by shifting to 36°C for 3.5 hours. They were then released at 25°C and time points taken every 15 minutes. The anti-microtubule drug carbendazim (CBZ) was added after 20 minutes. Extracts were made, Apc4-TAP pulled down with IgG Dynabeads, separated by SDS-PAGE and immunoblotted for associated Mad3p and Mad2p. B. The immunoblots were quantitated for Mad3p and Mad2p levels, normalised to Apc4p levels, and then plotted relative to the wild-type levels at the 45 and 60 minute time points (after release into mitosis). C. At each time point cells were fixed in 100% methanol and later stained with DAPI and calcofluor to score septation (200–300 cells were scored per sample, per time point). Septation indicates a failure to maintain the spindle checkpoint arrest.

Having detected only subtle MCC assembly defects in *mad3-C9A and mad3-C9A mad2-S92A*, we wanted to test the ability of the mutant MCC produced to bind to APC/C. These C-terminal phosphorylation sites lie close to the second KEN box in Mad3p (KEN2), which has been argued to be important for APC/C interactions in vertebrate cells [57]. Our previous work has shown that the first Mad3p KEN box (KEN1) is critical for Cdc20 ^Slp1^ binding and MCC assembly, and that whilst KEN2 is not required for MCC assembly it is needed for fission yeast checkpoint arrest [20]. To analyse this further, we carried out *cdc25* block and release time courses and monitored APC/C binding with the *mad3-KEN* mutants. The anti-microtubule drug carbendazim (CBZ) was added 20 minutes after *cdc25* release to test whether these *mad3* alleles could maintain a spindle checkpoint arrest. As expected, the *mad3-KEN1* allele displayed no APC/C binding, for either Mad3p or Mad2p (Fig 4). The *mad3-KEN2* allele displays comparable MCC-APC/C binding to wild-type Mad3p at early time points (45 minutes), but was unable to maintain MCC-APC/C levels or the checkpoint arrest (MCC-APC/C levels had already fallen to ~65% of wild-type levels at 60 minutes). We scored maintenance of checkpoint arrest by analysing when cells exit mitosis and septated during these time courses (Fig 4C). 50–60% of cells had septated by 2 hours in *mad3∆*, *mad3-KEN1* and *mad3-KEN2*. These findings are consistent with KEN2 not being required for MCC assembly or initial APC/C binding (at 45 minutes), but instead being needed to maintain APC/C binding. A vertebrate study has argued that the BubR1^Mad3^-KEN2 box can compete with securin/cyclin substrates and thereby prolong spindle checkpoint delays [57]. Another, more recent study proposed the involvement of the BubR1-KEN2 box in mediating interaction of a second molecule of Cdc20^Slp1^, bound to APC/C [58]. This latter model is currently being analysed in *S*.*pombe* (see Discussion).

**Fig 5:**
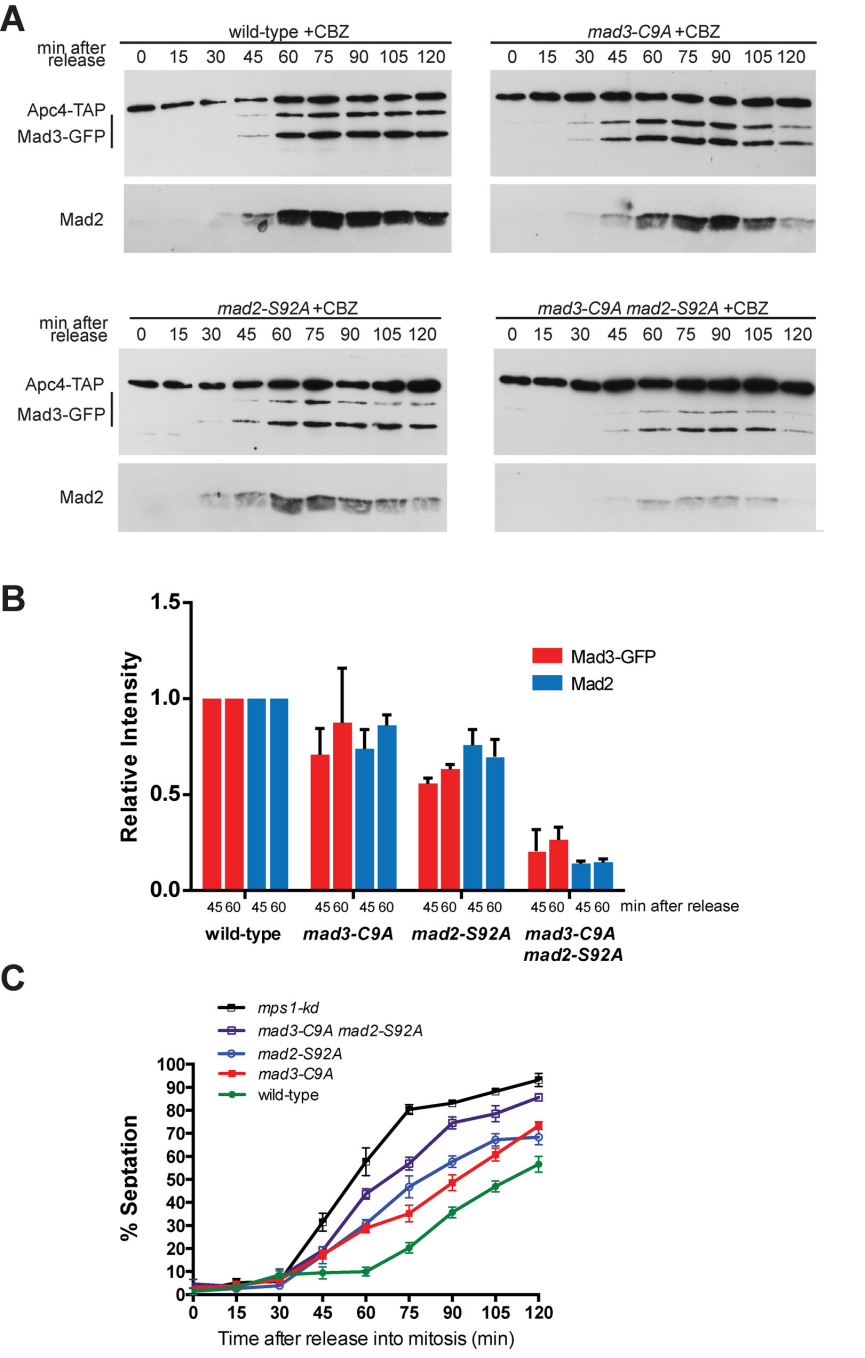
*mad3* and *mad2* phospho-mutants fail to maintain stable MCC-APC/C complexes. A. The *cdc25* strains indicated were pre-synchronised in G2 by shifting to 36°C for 3.5 hours. They were then released at 25°C and time points taken every 15 minutes. Carbendazim (CBZ) was added after 20 minutes. Extracts were made, Apc4-TAP pulled down with IgG Dynabeads, separated by SDS-PAGE and immunoblotted for associated Mad3p and Mad2p. B. The immunoblots were quantitated for Mad3p and Mad2p levels, normalised to Apc4p levels, and then plotted relative to the wild-type levels at the 45 and 60 minute time points (after release into mitosis). Plotted as mean with standard deviation (n = 2 experiments). C. Methanol-fixed cells were stained with calcofluor and scored for septation, indicating a failure to maintain spindle checkpoint arrest. This experiment was repeated twice and >100 cells scored for each strain at each time point. Plotted as mean with SEM.

To test whether mutation of the C-terminal Mad3p phospho-sites flanking KEN2 perturbed MCC-APC/C binding, we carried out *cdc25* (G2) block and release time courses and immunoprecipitated the APC/C from *mad3-C9A* mutants. This experiment demonstrates that the *mad3-C9A* and the *mad2-S92A* alleles displayed significantly reduced APC/C binding to Mad3p and Mad2p at 45 and 60 minutes (Fig 5A and 5B), ie. at times when rather little effect on MCC assembly was observed (see Fig 3B). The levels of Mad2p and Mad3p bound to the APC/C in *mad3-C9A mad2-S92A* were only 20% of those in wild-type at 45 and 60 minutes (Fig 5B). Importantly, at equivalent times in mitosis their MCC levels were 70–100% of those in wild-type cells (Fig 3B). We scored maintenance of checkpoint arrest by analysing when cells exit mitosis and septated during such time courses (Fig 5C). The *mad2-S92A* and *mad3-C9A* strains both septate ahead of wild-type, and the double mutant is even more advanced although not quite as fast as the *mps1-kd* strain (see S5 Fig for representative images and a direct comparison with the *mad3-KEN* mutants).

These data strongly support a model in which Mps1^Mph1^-dependent phosphorylation of both Mad2p and Mad3p are required to stabilise their APC/C binding and thus to maintain the MCC-APC/C complex and spindle checkpoint arrest.

### *In vitro* reconstitution confirms a direct role for Mad3p phosphorylation in APC/C inhibition

**Fig 6:**
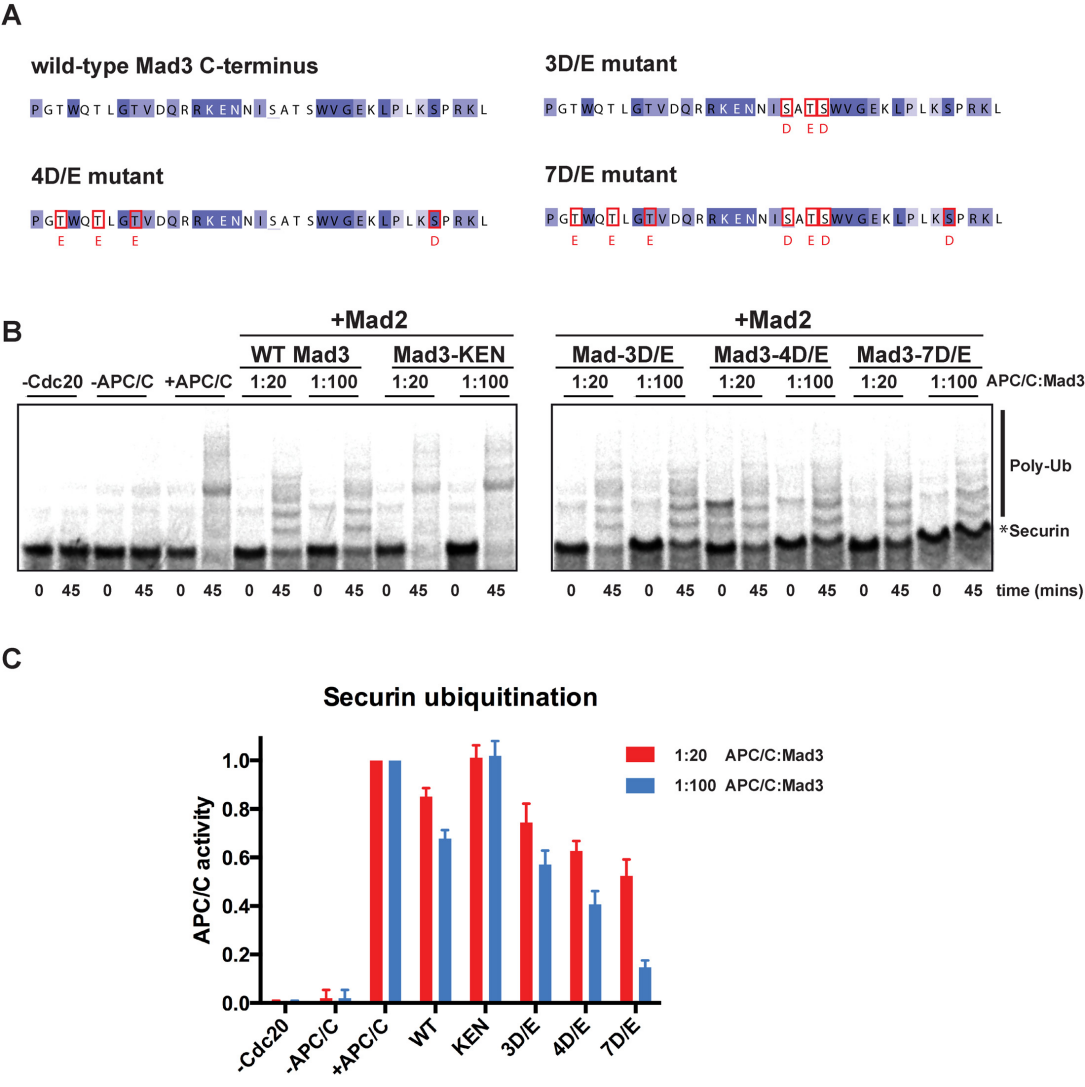
Mad3p modification *directly* affects its ability to inhibit APC/C activity. A. Mad3p C-terminal sequence highlighting the residues mutated in the 3, 4 and 7D/E mutants. B. Fission yeast APC/C *in vitro* activity reactions showing the ubiquitination of securin^Cut2^. Half of each reaction (10 μl) was taken immediately after mixing all the components and added to 4xSDS gel buffer (0 min). The remaining 10 μl were incubated at 23°C for 45 min. Control reactions, one lacking the activator Cdc20^Slp1^ (-Cdc20), one lacking the APC/C (-APC/C) and one containing both Cdc20^Slp1^ and APC/C (+APC/C), are shown together with wild-type Mad3p (WT), the double KEN box mutant (*mad3-KEN1*,*2-AAA)* and the corresponding phospho-mimic mutants at 1:20 and 1:100 APC/C:Mad3p molar ratios respectively. Purified cMad2p was added at 1:12.5 APC/C:Mad2p molar ratio in all reactions. A phosphoimager and Imagequant software were used to quantify the amount of radioactivity left in the un-modified securin band (*). C. Quantification of APC/C inhibition by wild-type (WT) Mad3p and mutants. Results from three independent experiments were combined and plotted (as mean with SEM). Data were normalised against APC/C activity containing no Mad2p or Mad3p (+APC/C).

Phosphorylation of Mad3p does not appear to regulate its kinetochore targeting (Fig 2A) or assembly into MCC (Fig 3). Even so, the effects we have observed on MCC-APC/C interactions (Fig 5) and checkpoint arrest (Fig 2B–2D) could be indirect consequences of a defective upstream signalling event. To confirm that Mad3p modification has a direct role in APC/C inhibition we developed an *in vitro* APC/C assay, based on those previously described for both budding and fission yeasts (see Materials and Methods, [59,60,61]). Fig 6 demonstrates that recombinant fission yeast Mad3p can directly inhibit *in vitro* Cdc20^Slp1^-APC/C activity: as the concentration of recombinant Mad3p was increased, a corresponding decrease in Cdc20 ^Slp1^-APC/C dependent ubiquitination of the radio-labelled securin^Cut2^ substrate was observed. Note this experiment was performed in the presence of Mad2p. Recombinant Mad3p inhibited APC/C activity on its own, but was approximately 30% less potent than the Mad3p-Mad2p combination. To test the effects of Mad3p phosphorylation on APC/C activity, we made a series of mutants containing phospho-mimics (S/D and/or T/E substitutions). These phosphorylation sites are in close proximity to the KEN2 box, suggested to be important for Mad3p-APC/C interaction and/or competing with substrates for APC/C binding (Fig 6A). We analysed their ability to directly inhibit Cdc20^Slp1^-APC/C activity *in vitro* (Fig 6B and 6C). The *mad3-double KEN* mutant protein (*KEN1*,*2-AAA*) was used here as a negative control. This experiment was repeated three times and Fig 6C shows quantitation of the combined data.

These experiments demonstrate that the Mad3 phosphomimics are better *in vitro* APC/C inhibitors than the recombinant wild-type Mad3 protein and that they display a graded increase in potency (c.f. 0,3,4 and 7 phospho-mimicking D/E residues). We conclude that mimicking phosphorylation of Mad3p near its C-terminal KEN2 box can directly improve its ability to inhibit Cdc20^Slp1^-APC/C activity.

### Mad3 phospho-mimics are not sufficient for APC/C inhibition in vivo

To test whether Mad3p modification *is sufficient* to induce a mitotic delay *in vivo*, we replaced wild-type *mad3* with alleles expressing 3, 4, 6 or 7D/E residues. These alleles produce stable proteins (see S7A Fig) that can be targeted to kinetochores in an apparently normal manner (S7B Fig). We released these strains from a *cdc25-ts* (G2) block into a synchronous mitosis, but no significant metaphase delay was observed. Further studies demonstrate that the *mad3-3D/E* and *-4D/E* alleles perform as well as wild-type in other checkpoint assays, but that they do not act as gain-of-function alleles (see S7C and S7D Fig for further details). Thus we conclude that such Mad3p modifications are not sufficient to induce and maintain a spindle checkpoint delay in cells.

## Discussion

Here we have identified Mad3p as an important substrate for Mps1^Mph1^ kinase, and demonstrated that its C-terminal modification is required for maintenance of spindle checkpoint arrests in response to microtubule (tubulin mutants or CBZ treatment, [Fig 2C and 2D and Fig 5]) and kinetochore defects (*nuf2-3* delays [Fig 2C]). In addition, our analysis of *mad3-C9A*,*mad2-S92A* double mutants (Figs 2, 3 and 5) suggests that Mps1^Mph1^ kinase modifies both Mad3p and Mad2p, and that these act in a concerted fashion to maintain robust inhibition of APC/C. *In vitro* ubiquitination assays confirm that modified forms of Mad3p (phospho-mimics) are more potent APC/C inhibitors than the unmodified protein. We propose that phosphorylation of the Mad3p C-terminus, in concert with Mad2p modification, stabilises its interactions with APC/C and thereby enhances the ability of MCC to inhibit APC/C and delay anaphase onset.

These studies have highlighted the fact that Mps1 ^Mph1^ kinase has multiple roles to play in spindle checkpoint signalling. Its activation at kinetochores may initiate the spindle checkpoint response [62] and several studies demonstrate that KNL1 is an important target. Once phosphorylated KNL1 recruits the Bub1-Bub3-Mad3 module [37,38]. Then Mps1^Mph1^ kinase phosphorylates Bub1p to stabilise a Mad1p interaction, at least in budding yeast [45]. This Bub1p-Mad1p complex may then be able to catalyse the formation of MCC [6,63]. However, we have shown here that this is not all that Mps1^Mph1^ kinase does in the checkpoint, as both Mad3p and Mad2p also need to be phosphorylated by Mps1^Mph1^ for cells to efficiently maintain APC/C inhibition and spindle checkpoint arrest. Thus Mps1^Mph1^ kinase has key roles to play in both the establishment and maintenance of spindle checkpoint arrests.

### Phosphorylation of Mad3/BubR1

The Mad3/BubR1 protein is a target of many mitotic kinases. Budding yeast Mad3p is a target of both Polo^Cdc5^ [64] and Aurora B^Ipl1^ kinases [65], the latter being required for cells to respond to lack of tension at kinetochores. In *Drosophila* it was reported that Polo promotes Mps1^Mph1^ kinetochore localisation and that this is required for BubR1 phosphorylation and stable MCC production [66]. In *Xenopus* egg extracts BubR1 is phosphorylated by Plx1, generating the 3F3/2 epitope, and this is required for checkpoint arrest [67]. However, in human cells BubR1 phosphorylation by Plk1 was shown to be important for regulating the stability of kinetochore-microtubule attachments [68]. The C-terminal KARD domain in BubR1, not present in yeast Mad3, is modified by both Polo and CDK kinases and this modification regulates recruitment of PP2A regulatory sub-units to kinetochores [69]. Once there, PP2A can oppose the action(s) of Aurora B and play a part in the onset of mitotic exit. Thus Mad3/BubR1 is a highly regulated phospho-protein, consistent with it being a key regulator of mitotic progression. However, the specific kinases involved and the mechanistic roles of Mad3/BubR1 phosphorylation vary between model systems.

Here we have demonstrated that fission yeast Mad3p is modified by several mitotic kinases: Mps1^Mph1^ and most likely CDK and Aurora^Ark1^. Our studies indicate that the C-terminal Mps1^Mph1^ sites flanking the Mad3p KEN2 box are particularly important, and that their modification can enhance the ability of this checkpoint protein to bind to and inhibit Cdc20^Slp1^-APC/C. Our *in vivo* studies of the phospho-mimic mutants (see S7 Fig) argue that these C-terminal Mad3p modifications are not sufficient to stabilise MCC-APC/C binding enough to induce a dominant mitotic delay. We propose that additional modifications, perhaps on Mad2p, Cdc20^Slp1^ or APC/C subunits would be needed to generate such a gain-of-function phenotype *in vivo*. In addition, cells probably have multiple checkpoint silencing pathways, not all of which would necessarily involve de-phosphorylation.

### Combinatorial control of MCC action

In addition to the C-terminus of Mad3p (this study), Mad2p (here and [39,70]) and Cdc20^Slp1^ [13,71,72] are phosphorylated and their modification is also thought to ensure efficient APC/C inhibition. Figs 2 and 5 demonstrate the synthetic defect of mutating Mad3p and Mad2p phosphorylation sites. Whilst the crystal structure of fission yeast MCC beautifully demonstrated the importance of the N-terminal KEN box and conserved TPR domain in MCC formation [18], this structure lacked the N-terminus of Cdc20^Slp1^ and the C-terminus of Mad3p. Both of these apparently unstructured regions are post-translationally modified and both are critical for *in vivo* regulation of Cdc20^Slp1^-APC/C activity (our study and [72]). It has recently been suggested that a second molecule of vertebrate Cdc20^Slp1^ might be involved in MCC-Cdc20^Slp1^-APC/C complexes [58]. We are currently assessing this model in fission yeast cells, but it is clear that further structural analyses employing recombinant, full-length Cdc20^Slp1^(s) and Mad3p will be needed before MCC interactions with Cdc20^Slp1^-APC/C can be fully understood.

## Materials and Methods

### Generation of Mad3 phosphorylation mutants

Phosphorylation mutants were created from pJK-Mad3, which is based on the vector pJK148 [73]. It contains 500 bp of 5’UTR, followed by the *mad3* ORF and 500 bp of 3’UTR. All *mad3* strains were generated by integration of pJK-Mad3 vectors into a *mad3Δ* strain at the endogenous *mad3* locus. Mad3 gene synthesis (GeneArt) was used to generate the 18A allele (see Fig 1). The C9A and N9A alleles were created from the 18A vector by sub-cloning, using an Xho1 site present in the Mad3 gene. All other point mutations were introduced using the Quikchange Kit for Site Directed Mutagenesis (Stratagene). For the APC/C binding assays C-terminal GFP tags were introduced using the Bahler cassette system (Bahler et al. 1998)

Recombinant wild-type (wt) and phospho-mimic Mad3 mutants as well as the closed Mad2 mutant (cMad2 [39]) used for the APC/C activity assays were constructed via Gateway cloning using the donor vector pDONR201 (Life technologies) and the destination vector pHMGWA described previously [74] containing 6xHis and Maltose Binding Protein (MBP) tags. Proteins were expressed in BL21 RIL cells with 0.25 mM IPTG at 18°C for 16hrs. Cells were harvested by centrifugation and pellets were frozen at -20°C until further use. Cells were lysed by sonication in lysis buffer A containing 50 mM potassium phosphate pH 7.0, 300 mM NaCl, 5% glycerol, 1mM DTT and 1% glucose supplemented with 1 mM Pefabloc (SIGMA Aldrich) and a cocktail protease inhibitor tablet (Roche). cMad2 and Mad3 proteins were affinity purified using amylose beads (NEB) and eluted with buffer A containing 15 mM maltose. Eluted fractions were pooled together and further purified by size exclusion chromatography using a Superdex S200 column (GE Healthcare) either in buffer B (for cMad2) containing 25 mM HEPES pH 7.5, 150 mM NaCl, or in buffer C (for Mad3 proteins) containing 25 mM potassium phosphate pH 7.0, 200 mM NaCl, 5% glycerol, 1mM DTT, 1% glucose and 1 mg/ml NVOY polymer (Expedeon). Protein purity was visualised by SDS-PAGE.

### Cdc20^Slp1^ FLAG tagging

First, the 3’ UTR of the Slp1^Cdc20^ gene (460 base pairs downstream of the stop codon) was marked with the Hygromycin B resistance gene through PCR based gene modification in yeast. Then the genomic DNA of the Slp1^Cdc20^ gene with this inserted Hyg B resistance marker was amplified by PCR and cloned into the Gateway® pDONR 201 vector (Life Technologies). A 5’ fragment of the Slp1^Cdc20^ gene (the first 450 base pairs), with an insertion of three tandem copies of the FLAG-tag between nucleotides 191 and 192, was commercially synthesised (Geneart® Life Technologies) and then cloned into the Slp1^Cdc20^ gene in the vector. The final construct was then linearised and used to transform haploid wild type yeast cells. Transformants were selected through their Hygromycin B resistance, and correct integration was confirmed by PCR and DNA sequencing. FLAG-Slp1^Cdc20^ expression was confirmed by immunoblotting.

### Fission yeast strains

Yeasts strains are listed in S1 Table.

### Mitotic arrests

For *nuf2-3* arrests, cells were grown overnight in YES medium at 25°C to mid-log phase and then shifted to 32°C for 4 hrs followed by fixation at -20°C in 100% methanol. For microscopy, cells were mounted with 20 μg/ml DAPI (Sigma).

*nda3-KM311*(cs) cells were grown overnight in yeast extract plus supplements (YES) medium at 30°C to mid-log phase and then shifted to 18°C for 6 hrs.

#### Preparation of the anti-microtubule drug carbendazim (CBZ)

A carbendazim stock solution (3.75 mg/ml) was prepared by dissolving it in DMSO. This solution was vortexed and heated in a 55°C water bath, either for 1 hr (for 65 μg/ml [Fig 2]) or 3 hrs (for 75 μg/ml [Fig 5]). Then it was vortexed again and cooled down to room temperature before use.

#### G2 arrests (cdc25-22) for synchronous mitotic analyses and CBZ challenge

*cdc25-22* cells were grown overnight in YES medium at 25°C to mid-log phase and then shifted to 36°C for 3.5 hrs to arrest cells in G2. To release into mitosis, cultures were rapidly cooled down to 25°C in iced-water and then incubated at 25°C for the experimental time course. 20 mins after the G2 release into mitosis, the CBZ solution was spun at room temperature for 1 min at 4500 rpm, and the soluble phase added to liquid cultures at 65 or 75 μg/ml final concentration.

### Benomyl sensitivity assay

10-fold serial dilutions were plated on YES plates containing 0, 7, 8.5 μg/ml of the microtubule depolymerising drug benomyl. Plates were incubated at 30°C for 3 days before images were taken.

### Immunostaining

10–50 ml of overnight culture were harvested and then fixed with -80°C cold methanol and washed twice with PEM (100 mM PIPES, pH 7.6, 1 mM MgSO_4_, 1 mM EGTA). Cell walls were then digested in PEMS (PEM, 1 M sorbitol) with 0.4 mg/ml Zymolyase (MP Bio-medicals) for 30–45 min. Cells were then sequentially washed once with PEMS, PEMS-TritonX-100 (1%) and PEM. Cells were blocked with PEMBAL (1% BSA, 0.1% L-Lysine in PEM) for 1 h and then incubated overnight with TAT1 (mouse anti-tubulin) antibody (1:50) (kindly provided by Keith Gull, Oxford, UK). Cells were washed once with PEM and then incubated with anti- mouse secondary antibody (Alexa Fluor–Molecular Probes) at 1:1000 for 1 h. Mitotic spindles were visualized using an Intelligent Imaging Innovations Marianas microscope (Zeiss Axiovert 200M, using a x100 1.3NA objective lens), CoolSnap CCD, and Slidebook software (Intelligent Imaging Innovations, Inc., Boulder, CO).

### Immunoprecipitations

#### Anaphase promoting complex interaction

Cells expressing TAP-tagged Lid1 (Apc4) (original strain kindly provided by Kathy Gould, Vanderbilt, USA) and Mad2p and Mad3p tagged with GFP from their endogenous loci were pre-synchronised in G2 via the *cdc25-22* mutation. Proteins were extracted in lysis buffer (50 mM HEPES pH 7.5, 75 mM KCl, 1 mM MgCl_2_, 1 mM EGTA, 0.1% TritonX-100, 1 mM sodium vanadate, 0.1 μM microcystin, 10 μg/ml LPC (leupeptin/pepstatin/chymostatin) and 1 mM pefabloc). Cells were resuspended in lysis buffer and bead-beat twice for 20 seconds. Extracts were incubated for 30 min with IgG-coupled Dynabeads (Invitrogen), which bind to Apc4-TAP. The immunoprecipitated complexes were washed three times with lysis buffer and then analysed by immunoblotting with sheep anti-GFP antibody and sheep anti-Mad2 antibody.

#### MCC immunoprecipitation

Cdc20^Slp1^-FLAG was immunoprecipitated as above, but using anti-FLAG (M2, Sigma) antibodies that had been pre-coupled to Dynabeads (Invitrogen).

Quantitation of relative levels of MCC complexes:

For each strain and time point an equivalent cell pellet was lysed using the same volume of glass beads and lysis buffer per sample. All samples for any figure panel were processed at the same time and the full set of IPs were run on gels in parallel (whole cell lysates were typically run the following day). eg. membrane organisation for Fig 3: wild-type & *mad3-C9A* IPs were on one membrane, *mad2-S92A *& *mad3-C9A mad2-S92A* IPs on another, *mps1-kd* on a third. The input lysates were run and transferred to membranes as for the IPs.

Membranes for all IP samples were incubated with primary antibody, then secondary antibody and then HRP substrate at the same time. These incubations (eg. anti-FLAG) were carried out in the same box for all 3 membranes. Note, the same membrane was first probed with anti-FLAG (Cdc20^Slp1^) and anti-Mad2, then stripped and re-probed with anti-GFP (Mad3), so one pipetted IP sample was being analysed for all 3 proteins.

For each antibody all of the membranes were exposed at the same time to a single piece of film for each exposure. The exposure times used gave signal intensities within the linear range, as determined by serial dilution of lysates. Relatively short exposures were used to avoid saturation (e.g. 60 secs for anti-FLAG and for anti-Mad2). Band intensities were measured using ImageJ and corrected for background. Where relevant, degradation products were combined in the quantification (we also quantified the 2 bands separately, and this essentially gave the same result).

The relative level of an MCC sub-unit in the complex, compared to that of the immunoprecipitated protein (FLAG-tagged Cdc20) were then determined for the 45 and 60 minute time points for each strain, as these time points are before cells exit mitosis and Cdc20 levels drop. Eg.

$$\frac{\mathrm{Mad}2\;\left(\mathrm{or}\;\mathrm{Mad}3\right)\;\mathrm{band}\;\mathrm{intensity}}{\mathrm{Cdc}20^{\mathrm{Slp}1}\;\mathrm{band}\;\mathrm{intensity}}=\mathrm{relative}\;\mathrm{intensity}\;\mathrm{of}\;\mathrm{Mad}2\;\left(\mathrm{or}\;\mathrm{Mad}3\right)$$

Then we calculated the relative level of each sub-unit in the mutant compared to that in the wild-type strain:

$$\frac{\mathrm{Relative Mad}2\;\left(\mathrm{or Mad}3\right)\;\mathrm{intensity in the mutant}}{\mathrm{Relative Mad}2\;\left(\mathrm{or Mad}3\right)\;\mathrm{intensity in WT}}$$

and finally this was plotted as a % (eg. Fig 3B). Similar calculations were performed to determine the relative abilities of strains to form MCC-APC/C complexes, by comparing the levels of Mad2 and Mad3-GFP in Apc4-TAP pull downs (Fig 5). Error bars represent the mean with standard deviation.

#### Mps1^Mph1^-SZZ kinase purification

Cycling cells expressing Mph1-SZZ were pelleted, washed once with ice-cold water, frozen and ground in liquid nitrogen. The frozen cell powder was resuspended 2 x Hyman lysis buffer (100 mM Bis-Tris propane, 200 mM KCl, 10 mM EGTA, 10 mM EDTA, 20% glycerol, 1% TritonX-100 supplemented with protease inhibitors (1 mM pefabloc, 10 μg/ml leupeptin/pepstatin/chymostatin), and sonicated for 30 sec. Lysed cells were centrifuged at 4°C for 10 min at 3000 g to remove cell debris. The remaining supernatant was then sequentially filtered through a 2.6 μm and a 1.6 μm syringe filter. The clarified lysate was incubated with IgG-coupled dynabeads (Invitrogen) for 30 min at 4°C. Beads were washed three times with 1 x Hyman buffer and then three times with 1 x Hyman buffer (+ 1 mM DTT and 0.1% Tween-20). Bound proteins were eluted by incubating the beads with 100 units AcTEV protease (Invitrogen) overnight at 4°C. The supernatant was then transferred to a new tube and the IgG-coupled Dynabeads washed once with 1 ml 1 x Hyman buffer (+ 1 mM DTT, 0.1% Tween-20). The fractions containing the eluted proteins were combined and incubated with S-protein agarose beads (Novagen) for 3 hrs at 4°C. S-protein agarose beads bound to Mph1-SZZ were then washed five times with 1 x Hyman buffer and frozen at -80°C until further use.

### *In vitro* kinase assay

Purified Mps1^Mph1^ kinase coupled to S-protein agarose beads was washed twice with 1 x kinase buffer (50 mM Hepes pH 7.5, 10 mM MgCl_2_, 0.5 mM DTT). 25 μl of kinase reaction buffer (12.5 μl 2x kinase buffer (100 mM Hepes, pH7.5, 20 mM MgCl_2_, 1 mM DTT), 0.5 μl P^32^ gamma-ATP, 0.5 μl 1 mM ATP, made up with substrate in a final volume of 25 μl was then added to the beads. Reactions were typically carried out with 1.5 μg of substrate/recombinant protein. The reaction was incubated at 30°C for 30 minutes. Cold kinase assays were carried out with 100 mM ATP and further analysed by mass spectrometry.

### Mass-spectrometry

#### Protein digestion and Phosphopeptide enrichment

Proteins were electrophoresed into Novex NuPAGE 4–12% Bis-Tris gels (Invitrogen) and visualised with colloidal blue staining kit (Invitrogen). Proteins were excised, reduced/alkylated and in-gel digested using trypsin as described previously [75]. Peptides for direct MS analysis were desalted using C18 StageTips [76]. Peptides for phosphopeptide enrichment were extracted from the gel using 3% TFA/30% ACN followed by 100% ACN. Before enrichment approximately 3 mg TiO_2_ beads were pre-incubated in 20 μl of 85 mg/ml lactic acid in 80% ACN/0.1%TFA. This pre-incubated bead mixture was added to the peptide mixture and incubated for 1 h at room temperature [77]. After washing once with 10% ACN/0.1% TFA, and twice with 80% ACN/0.1% TFA, peptides were eluted with 2% ammonium hydroxide in 40% ACN (pH 10.5). Eluate was concentrated to 100 μl and 100 μl of 2% TFA was added and peptides desalted using C18 StageTips.

### Nano-LC-MS/MS and data analysis

Peptides were loaded onto a column needle self-packed [78] with ReproSil-Pur C18-AQ material (3 μm, Dr. Maisch, Germany) at a flow rate of 0.7 μl/min. Peptides were separated using an Aligent 1100 binary nanopump LC, with HTC Pal Auto sampler (CTC), using either a two-step linear gradient of 0%-20% B in 35 min, 20%-80% B in 4 min and 80% B for 2 min or a two-step linear gradient of 0%-20% B in 75 min, 20%-80% B in 13 min and 80% for 10 min (Mobile phases were (A) 5% acetonitrile, 0.5% acetic acid and (B) 99.5% acetonitrile, 0.5% acetic acid). Peptides were eluted into an LTQ-Orbitrap mass spectrometer (Thermo Fisher Scientific) using a flow rate of 300 nl/min and a spray voltage of 1.8kV.

### Database searching

DTAsupercharge (V1.18) was used to create peak lists from raw data. Peak lists were then used within Mascot daemon (V2.2.0) to search against the UniProt/SwissProt *Schizosaccharomyces pombe* database. Search parameters were set to: precursor mass tolerance of 10 ppm, fragment ion mass tolerance to 0.8 Da, enzyme as trypsin, allowing 3 missed cleavages. Carboamidomethylation of cysteine was set as a fixed modification, with oxidation of methionines, phosphorylation of serine, threonine, and tyrosine as variable modifications.

### Large scale yeast Mad3-SZZ and Lid1/Apc4-ZZ pull downs and MudPIT

Six litres of yeast cells were grown at 30°C in concentrated rich medium to high densities that could still support active proliferation and mitotic arrests. Mitotically arrested cells (harbouring cold-sensitive β-tubulin alleles) were obtained by rapid cooling on iced-water and shifting cultures to 18°C for 7 hours. Cells were harvested by centrifugation at 3,500g for 8 minutes at 4°C. Pelleted cells were frozen into pea-sized drops using liquid nitrogen and stored at -80°C until further processing. Approximately 25 g of cell mass was disrupted using a mixing mill (MM 400; Retsch, Germany) with grinding balls under cryogenic conditions (5 cycles of 3 minutes at 30 Hz). Yeast lysates were reconstituted in lysis buffer A containing 50mM Bis-Tris Propane-HCl pH 7.6, 10% (v/v) glycerol, 100 mM KCl, 5 mM EGTA and 1% Triton X100 supplemented with a ‘complete EDTA-free protease inhibitor’ tablet (per 50 ml), 1mM Pefabloc SC, 10 μg/mL leupeptin/pepstatin/chymostatin (all from Roche), 20 mM β-glycerophosphate, 5mM NaN_3_, 0.4 mM Na_3_VO_4_ and 2 μM microcystin-LR (Axxora Life Sciences, CA, USA). Following further lysis, by sonication, cell debris was pelleted at 4,000g for 5 min at 4°C and the supernatant was filtered through a 1.6 μm syringe filter. Proteins were bound to IgG-Dynabeads by incubating the cleared lysates for 20 min at 4°C (1 mg IgG-Dynabeads per 1 g of cell input) whilst mixing. Dynabeads with bound proteins were then washed four times with buffer A and twice with buffer B [50 mM HEPES-KOH pH 7.0, 50 mM KCl].

### Elution and precipitation of protein complexes

Proteins were eluted from Dynabeads by two x 5 min incubations with 150 μl of 100mM glycine-HCl (pH 2.5) at room temperature and precipitated with 100 μl of cold 100% (w/v) trichloroacetic acid. Proteins were pelleted at 4°C by centrifugation for 30 min at 22,000g. Supernatant was removed and pellets were washed twice with 500 μl of ice-cold acetone. and air-dried.

### LC/LC-MS/MS

Proteins were reduced, alkylated and digested with trypsin. Peptides were pressure vessel loaded onto a biphasic (C18-SCX) column. Peptides were separated by MuDPIT (Washburn et al., 2001) using an Aligent 1100 quaternery LC with a 1:1000 split flow system. Peptides were eluted into an LTQ mass spectrometer (Thermo Fisher Scientific) at spray voltage of 2.6 kV. Eight data-dependant ms/ms were collected per primary scan. MS3 scans were triggered with neutral loss values of 32.50, 49.00 and 98.00.

### APC/C ubiquitination assays

Fission yeast genes E1 (Uba1) and E2s (Ubc1, 4 and 11) inserted in pMAL and pET16b plasmids respectively were kindly provided by Hiro Yamano (Ors et al., 2009). These were used to recombinantly express proteins in BL21 RIL cells as described above for the Mad3 constructs. MBP-Uba1 and His-tagged E2s were then affinity purified using either amylose (NEB) or talon resin (Clonetech). MBP-Uba1 was subjected to further purification by size exclusion chromatography using a Sephadex S200 column. Full-length APC/C activator Cdc20 ^Slp1^ and radiolabelled [Met S^35^] APC/C substrate, Securin^cut2^ were generated by *in vitro* translation using the TNT Quick Coupled Transcription/Translation kit (Promega) according to manufacturer’s instructions using 1μg of pHY22-Cdc20^Slp1^ and pHY22-Securin^cut2^ plasmids also provided by Hiro Yamano. Lid1-TAP APC/C was affinity purified using IgG agarose beads (GE Healthcare) as previously described [59,79] from S. *pombe* cells carrying the *ts slp1-362* mutation [80] to allow the isolation of the APC/C free from endogenous Cdc20^slp1^.

*In vitro* APC/C ubiquitination assays were performed following a method based on assays previously described for budding, fission yeast and human proteins [59,61,81]. In brief, 0.05 mg/ml Uba1, 0.5mg/ml E2s (ubc1, ubc4 and ubc11) and 0.3 mM ATP were mixed with 1.5 mg/ml wild-type mono-ubiquitin (Sigma Aldrich), 2μM ubiquitin aldehyde (Boston Biochem) in a ubiquitination buffer containing 25 mM HEPES pH 7.5, 100 mM KCl, 3mM ATP, 2.5 mM MgCl_2_, and 0.2 mM DTT. The ubiquitination mix was incubated at 23°C for 20 min. 0.5 μM APC/C, 4 ul and 2 ul of Cdc20^Slp1^ and Securin^Cut2^ respectively were then added to the mix in a total reaction volume of 20 μl. Reactions were carried out at 23°C for 45 min and stopped by adding 4xSDS gel buffer. Samples were subjected to SDS-PAGE electrophoresis and visualised by radiography using the Typhoon phosphoimager. The ubiquitination of Securin^Cut2^ by APC/C was analysed using the ImageQuant software (GE Healthcare) by quantifying the decrease in intensity of the unmodified Securin band (labelled “*Securin” in Fig 6B). The ability of Mad3 and phospho-mimic mutants to inhibit the activity of APC/C was assessed by pre-incubating APC/C, Cdc20^Slp1^ and cMad2 with the corresponding Mad3 protein at 4°C for 30 min prior to adding them to the ubiquitination mix.

## Supporting Information

S1 Fig **(A) Fission yeast Mad3p alignments**. These fission yeast sequences are available at the Broad Institute (MIT, Harvard). The two sets of mutations (generating N9A and C9A) are highlighted on the *S*. *pombe* sequence with red boxes. T82 (a putative Aurora site) is in green. (B) Phosphosites identified. The superscript indicates the number with number of unique spectra/peptides in which the phosphorylated residue was identified. The residues highlighted in red (identified *in vitro* and confirmed *in vivo*) were only observed in mitotic samples.
(PDF)

S2 Fig Example spectra for Mad3 T259, T278 and S279. These serve as examples of MS/MS spectra used to identify and assign phosphorylation of peptides. Fragment ions containing the peptide’s N- (b-ions) or C- (y-ions) termini are labelled. A number of the identified peptides show neutral loss of phosphoric acid from the full-length peptide, supporting the conclusion of peptide phosphorylation. Significant fragmentation has also occurred allowing peptide sequencing and assignment of the phosphorylated residue.
(PDF)

S3 Fig *mad3-S289A* is not sensitive to anti-microtubule drugs. (A) Strains were plated on rich (YES) media containing the indicated concentrations of the microtubule disrupting drug benomyl, and grown at 30°C for 3 days. (B) *cdc25* strains containing wild-type *mad3*, *or mad3-S289A*, or *mps1-kd* mutations were synchronised in G2, released in mitosis and then challenged to arrest in response to 75μg/ml of the anti-microtubule drug carbendazim (CBZ). Failure to maintain mitotic checkpoint arrest leads to septation. This was scored at 15 minute time-intervals, by methanol-fixing cells and staining with calcofluor. The *mad3-S289A* allele behaved like wild-type.
(PDF)

S4 Fig Septation of *mad3* phosphomutants indicates defects in maintenance of spindle checkpoint arrest. (A) *cdc25* strains containing *mad3-C9A*, *mad2-S92A*, *mad3-C9A* and *mad2-S92A*, or *mps1-kd* mutations and GFP-tagged Mad3 were synchronised in G2, released in mitosis and then challenged to arrest in response to the anti-microtubule drug carbendazim (CBZ). Failure to maintain mitotic checkpoint arrest leads to septation. This was scored at 15 minute time-intervals, by methanol-fixing cells and staining with calcofluor. These images are from the 60 minute time-point and accompany the experiment presented in Fig 2D. The scale bar is 5 microns. (B) In some of the *mad3-C9A* cells the Mad3-GFP can still be detected on kinetochores after cells have septated. This observation is consistent with such cells failing to maintain their checkpoint arrest due to a “downstream” defect in APC/C binding, rather than a Mad3 defect in checkpoint signalling at kinetochores and/or the kinetochore attachments being satisfied in these mutant strains.
(PDF)

S5 Fig Septation of *mad3-KEN* and phosphomutants revealing defects in the maintenance of spindle checkpoint arrest. *cdc25* strains indicated were pre-synchronised in G2 by shifting to 36°C for 3.5 hours. They were then released at 25°C and time points taken every 15 minutes. Carbendazim (CBZ) was added after 20 minutes. Cells containing Mad3-GFP were fixed in methanol, stained with calcofluor and then scored for septation, which indicates a failure to maintain spindle checkpoint arrest. The images are from the 60 min time point, and represent the strains used in Figs 4 and 5. The scale bar is 5 microns.
(PDF)

S6 Fig Quantitation of Cdc20^Slp1^ levels in the cell extracts from Fig 3. The peak level of Cdc20^Slp1^ is ~80% in the *mps1-kd* strain when compared to wild-type in this and similar experiments. This can partly account for the reduced levels of MCC in *mps1-kd*, but not in the *mad3* phospho-mutants.
(PDF)

S7 Fig **(A) The recombinant Mad3-MBP fusions are all soluble and stable**. The gel shown here was stained with coomassie. (B) Mad3 phospho-mimics are targeted to kinetochores. The indicated strains were synchronised in G2 using the *cdc25* block, and then released into mitosis before imaging. Scale bar is 10 microns. (C) The *mad3-3D/E and 4D/E phospho-mimic* alleles are not significantly benomyl sensitive, but the *mad3-6D/E* and *7D/E* alleles are sensitive. Strains were plated on rich (YES) media containing the indicated concentrations of the microtubule disrupting drug benomyl, and grown at 30°C for 3 days. (D) The *mad3-3D/E and 4D/E phospho-mimic* alleles are checkpoint competent, but the *mad3-6D/E* and *7D/E* alleles are unable to maintain an *nda3* arrest. *nda3* strains containing the indicated *mad3* alleles, or the *mad2* deletion as a control, were shifted to 18°C for 4 and 6 hours. Plo1-GFP, which binds SPBs in mitosis, was used to score the mitotic index. The *mad3-3D/E* and *-4D/E* alleles arrest well. The *mad3-6D/E* and *-7D/E* alleles initially arrest (4 hours) but are unable to maintain the checkpoint arrest and by 6 hours have a significant defect. This indicates that having six or more constitutive phospho-mimic mutations near KEN2 perturbs Mad3p function.
(PDF)

S1 Table Fission yeast strain list. (PDF)
