# Correction: Mps1^Mph1^ Kinase Phosphorylates Mad3 to Inhibit Cdc20^Slp1^-APC/C and Maintain Spindle Checkpoint Arrests

**DOI:** 10.1371/journal.pgen.1006009

**Published:** 2016-04-21

**Authors:** Judith Zich, Karen May, Konstantinos Paraskevopoulos, Onur Sen, Heather M. Syred, Sjaak van der Sar, Hitesh Patel, James J. Moresco, Ali Sarkeshik, John R. Yates, Juri Rappsilber, Kevin G. Hardwick

The following information is missing from the Data Availability section: The *in vivo* mass spectrometry proteomics data have been deposited at the public data repository at UC San Diego (http://massive.ucsd.edu/ProteoSAFe/status.jsp?task=b6dc61704cc94f35919a16bb896a9424). The *in vitro* mass spectrometry proteomics data have been deposited at the ProteomeXchange Consortium via the PRIDE partner repository with the dataset identifier PXD003806 (http://www.ebi.ac.uk/pride/archive/projects/PXD003806).
