# Supplementary material for: Mps1Mph1 Kinase Phosphorylates Mad3 to Inhibit Cdc20Slp1-APC/C and Maintain Spindle Checkpoint Arrests
Source: PLoS Genet. 2016 Feb 16;12(2):e1005834. doi: 10.1371/journal.pgen.1005834 (PMC4755545; doi:10.1371/journal.pgen.1005834)
Supplement: S1 Fig — (A) Fission yeast Mad3p alignments. These fission yeast sequences are available at the Broad Institute (MIT, Harvard). The two sets of mutations (generating N9A and C9A) are highlighted on the S. pombe sequence with red boxes. T82 (a putative Aurora site) is in green. (B) Phosphosites identified. The superscript indicates the number with number of unique spectra/peptides in which the phosphorylated residue was identified. The residues highlighted in red (identified in vitro and confirmed in vivo) were only observed in mitotic samples. (PDF) [file pgen.1005834.s001.pdf]

A

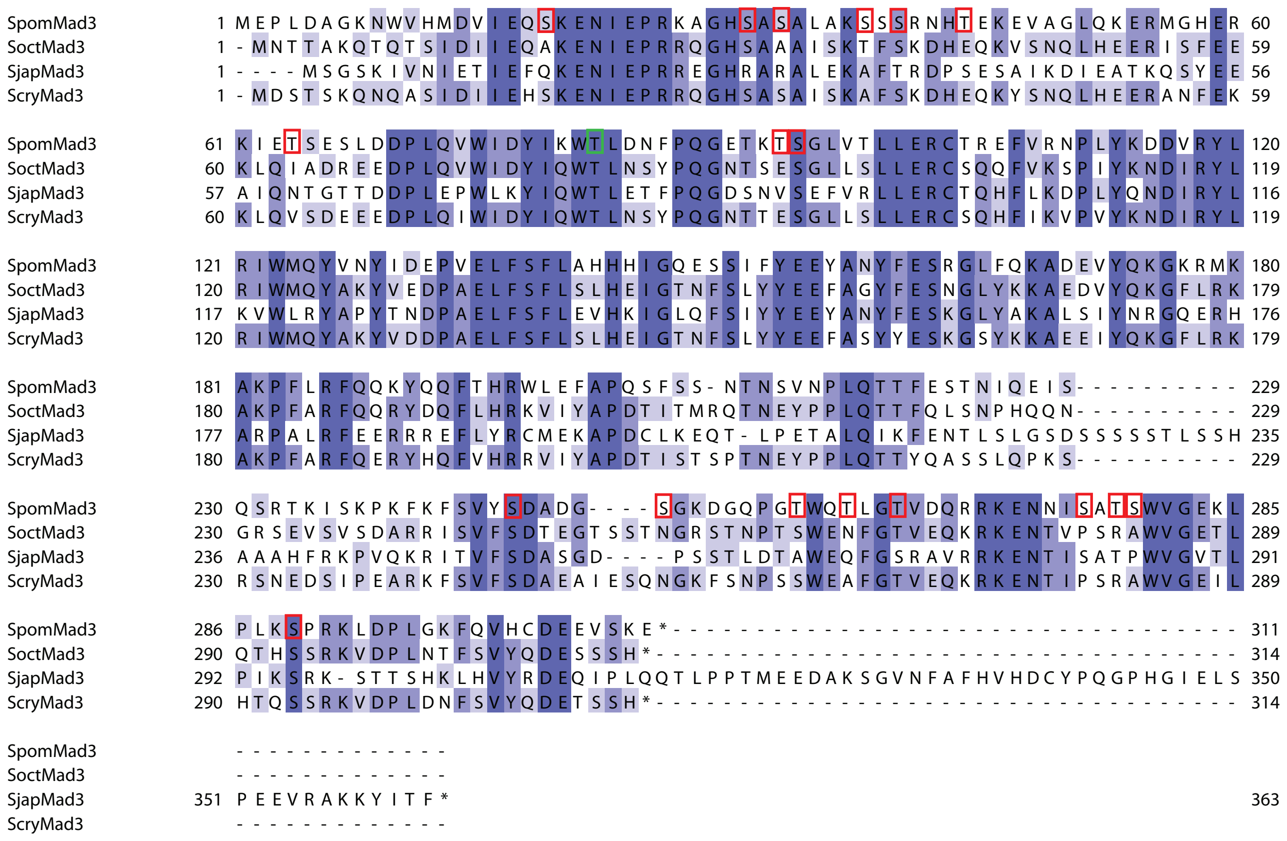

B

Mad3 phospho-sites

in vitro Mps1 kinase assay

S19,S31,S33,S38,S40,T44,T64,
T93,S94,T195,T259,T262,T265
S276,T278,S279

in vivo Mad3-TAP

S19,S31,S33[2]*,T64*[2]*,*
T82[3(Ark1?)]*,S246*[2]*,S251*[3]*,T259*[2]
S276[3]*,T278,S279,* ***S289***[10(CDK)]

in vitro Mps1 sites confirmed *in vivo*

S19,S31,S33,T64,
T259,S276,T278,S279

S1 Zich et al
