# Supplementary figures and images for: Mps1Mph1 Kinase Phosphorylates Mad3 to Inhibit Cdc20Slp1-APC/C and Maintain Spindle Checkpoint Arrests
Source: PLoS Genet. 2016 Feb 16;12(2):e1005834. doi: 10.1371/journal.pgen.1005834 (PMC4755545; doi:10.1371/journal.pgen.1005834)

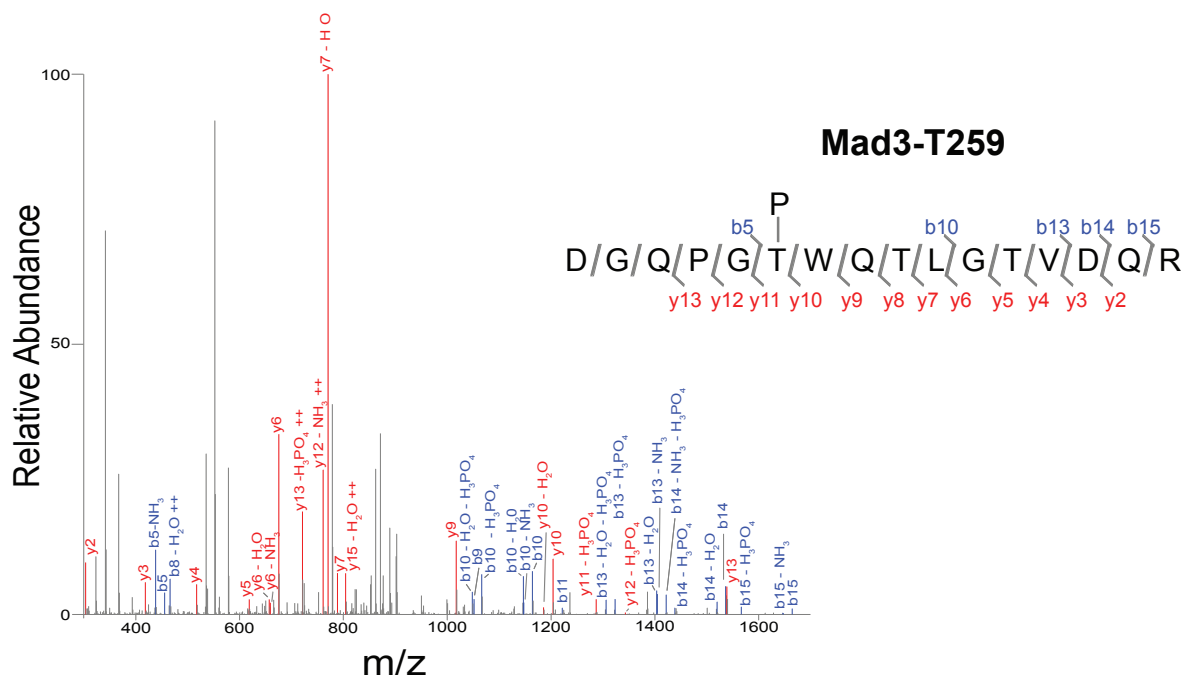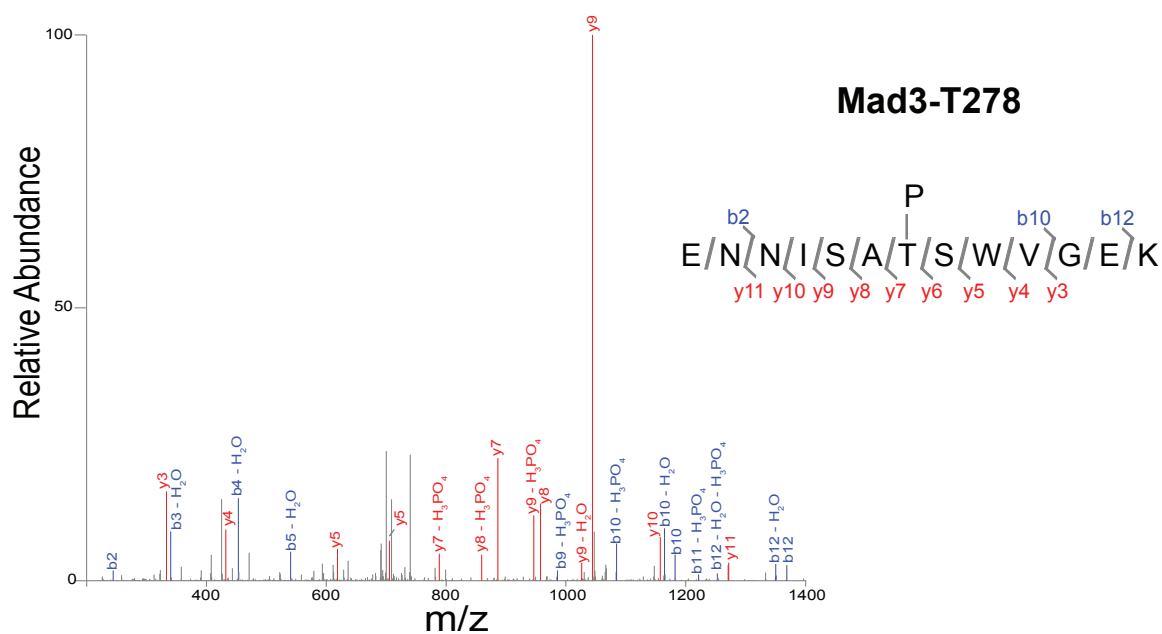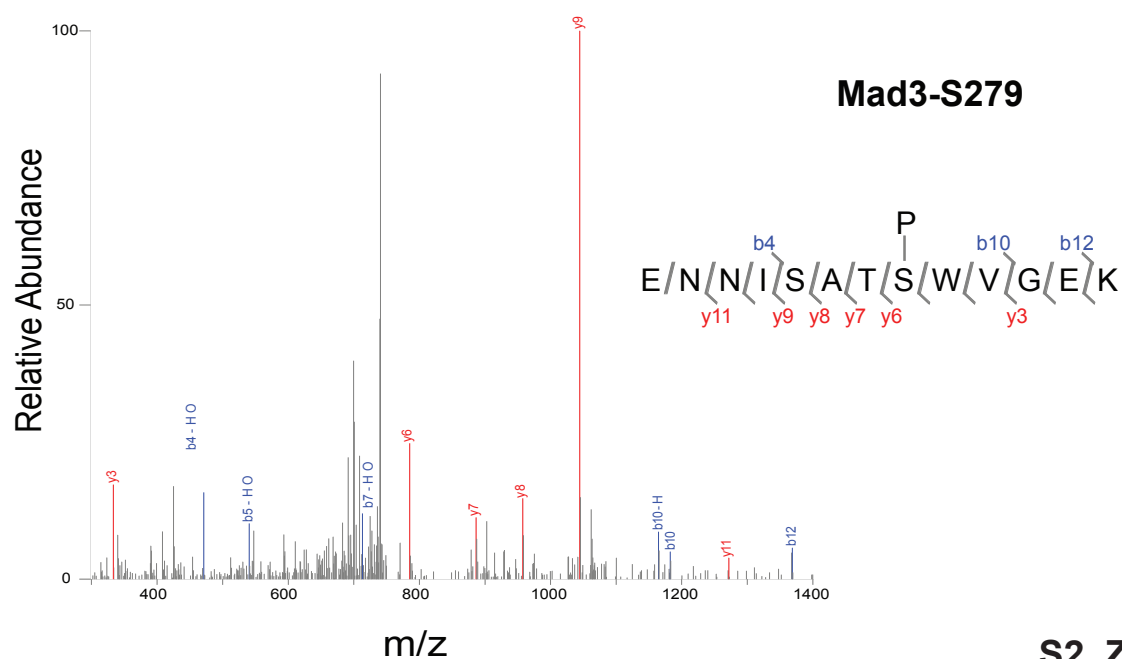

Supplement: S2 Fig — These serve as examples of MS/MS spectra used to identify and assign phosphorylation of peptides. Fragment ions containing the peptide’s N- (b-ions) or C- (y-ions) termini are labelled. A number of the identified peptides show neutral loss of phosphoric acid from the full-length peptide, supporting the conclusion of peptide phosphorylation. Significant fragmentation has also occurred allowing peptide sequencing and assignment of the phosphorylated residue. (PDF) [file pgen.1005834.s002.pdf]

**A**

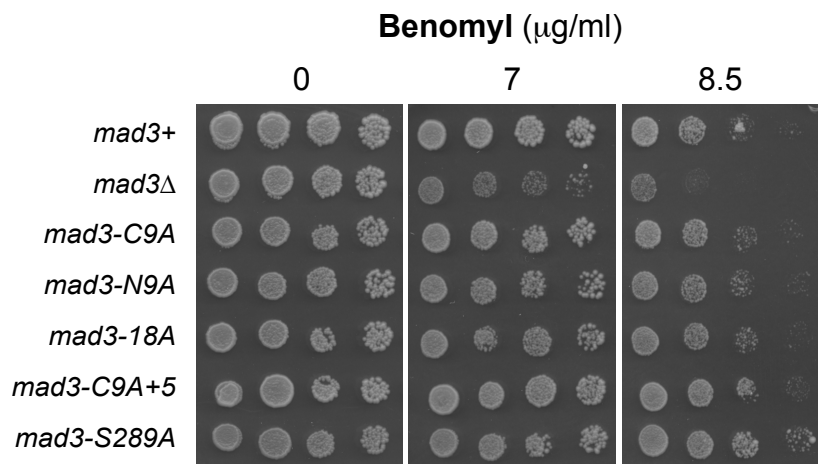

**B**

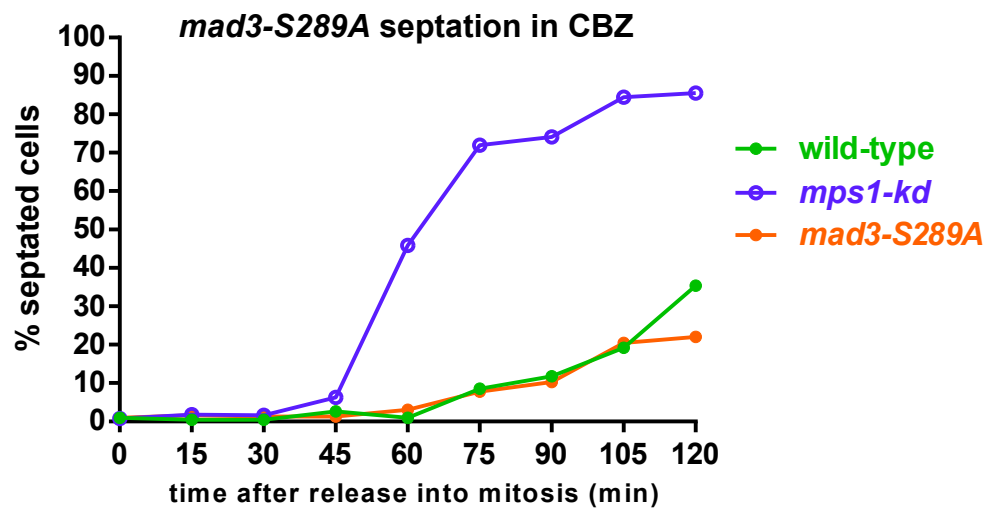

Supplement: S3 Fig — (A) Strains were plated on rich (YES) media containing the indicated concentrations of the microtubule disrupting drug benomyl, and grown at 30°C for 3 days. (B) cdc25 strains containing wild-type mad3, or mad3-S289A, or mps1-kd mutations were synchronised in G2, released in mitosis and then challenged to arrest in response to 75μg/ml of the anti-microtubule drug carbendazim (CBZ). Failure to maintain mitotic checkpoint arrest leads to septation. This was scored at 15 minute time-intervals, by methanol-fixing cells and staining with calcofluor. The mad3-S289A allele behaved like wild-type. (PDF) [file pgen.1005834.s003.pdf]

A

60 mins after release

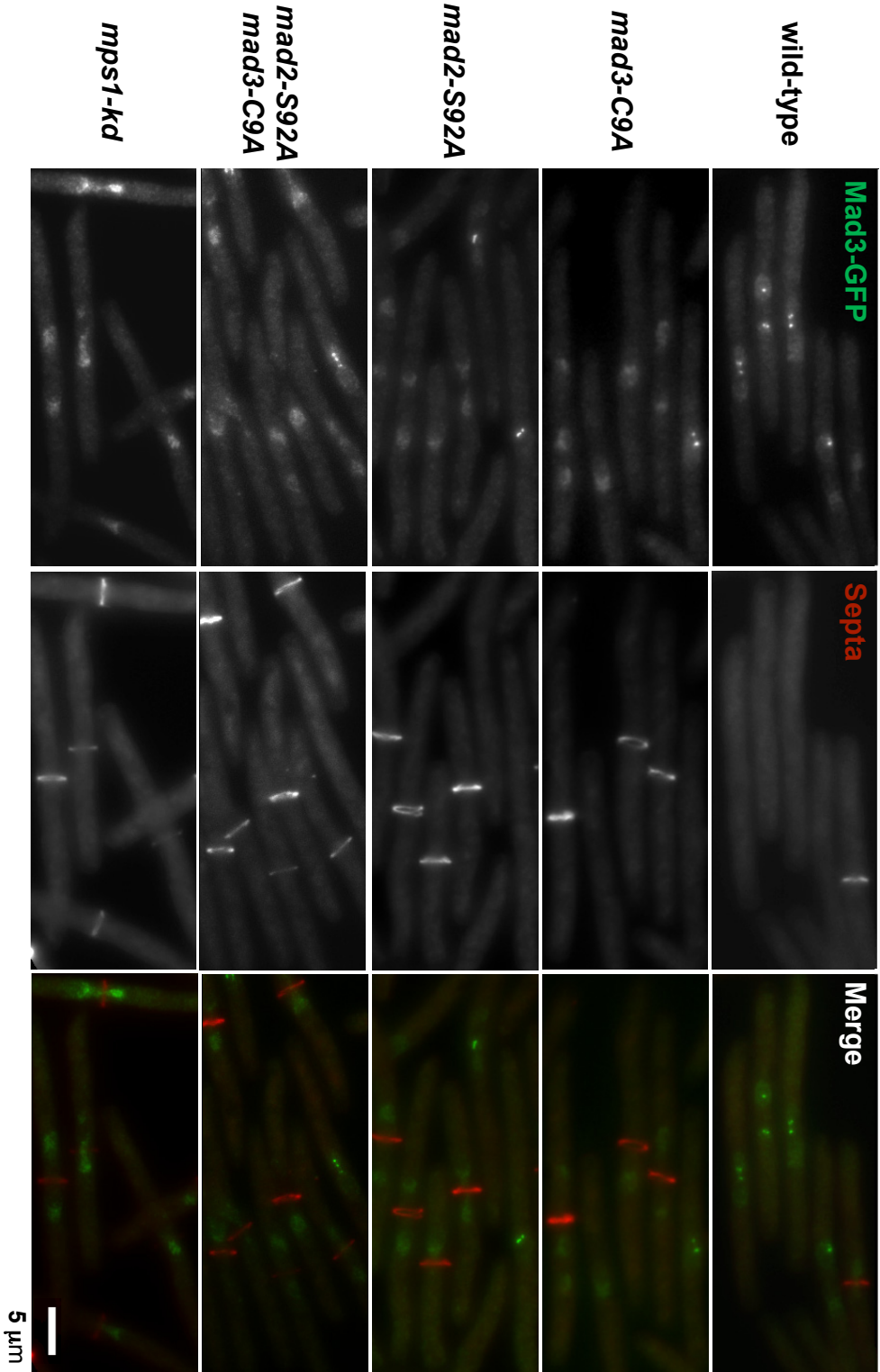

B

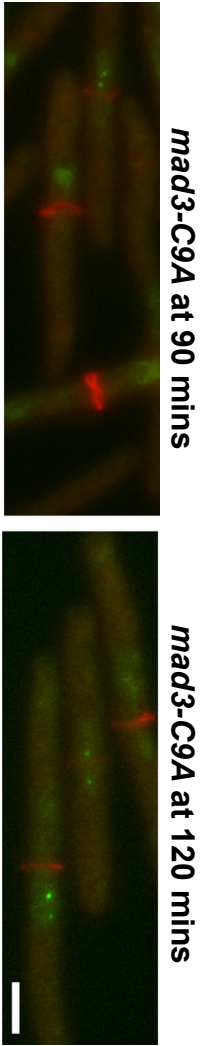

Supplement: S4 Fig — (A) cdc25 strains containing mad3-C9A, mad2-S92A, mad3-C9A and mad2-S92A, or mps1-kd mutations and GFP-tagged Mad3 were synchronised in G2, released in mitosis and then challenged to arrest in response to the anti-microtubule drug carbendazim (CBZ). Failure to maintain mitotic checkpoint arrest leads to septation. This was scored at 15 minute time-intervals, by methanol-fixing cells and staining with calcofluor. These images are from the 60 minute time-point and accompany the experiment presented in Fig 2D. The scale bar is 5 microns. (B) In some of the mad3-C9A cells the Mad3-GFP can still be detected on kinetochores after cells have septated. This observation is consistent with such cells failing to maintain their checkpoint arrest due to a “downstream” defect in APC/C binding, rather than a Mad3 defect in checkpoint signalling at kinetochores and/or the kinetochore attachments being satisfied in these mutant strains. (PDF) [file pgen.1005834.s004.pdf]

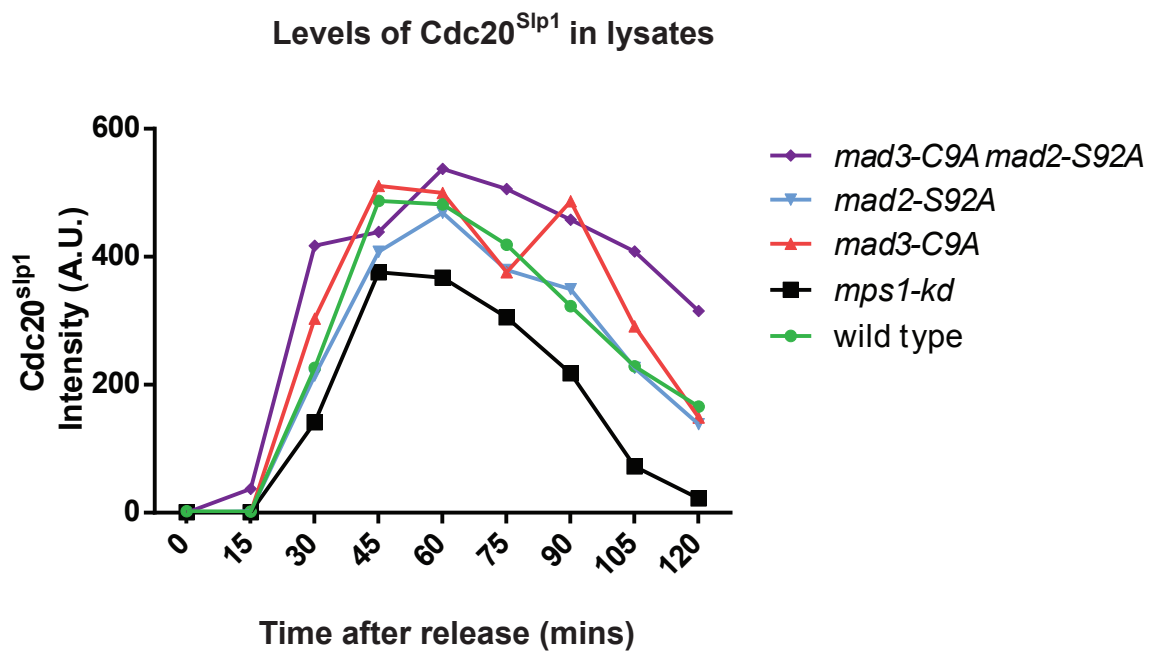

S6 Zich et al.

Supplement: S6 Fig — The peak level of Cdc20Slp1 is ~80% in the mps1-kd strain when compared to wild-type in this and similar experiments. This can partly account for the reduced levels of MCC in mps1-kd, but not in the mad3 phospho-mutants. (PDF) [file pgen.1005834.s006.pdf]

**A**

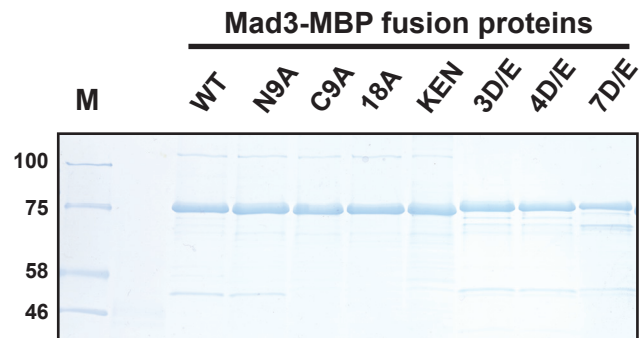

**B**

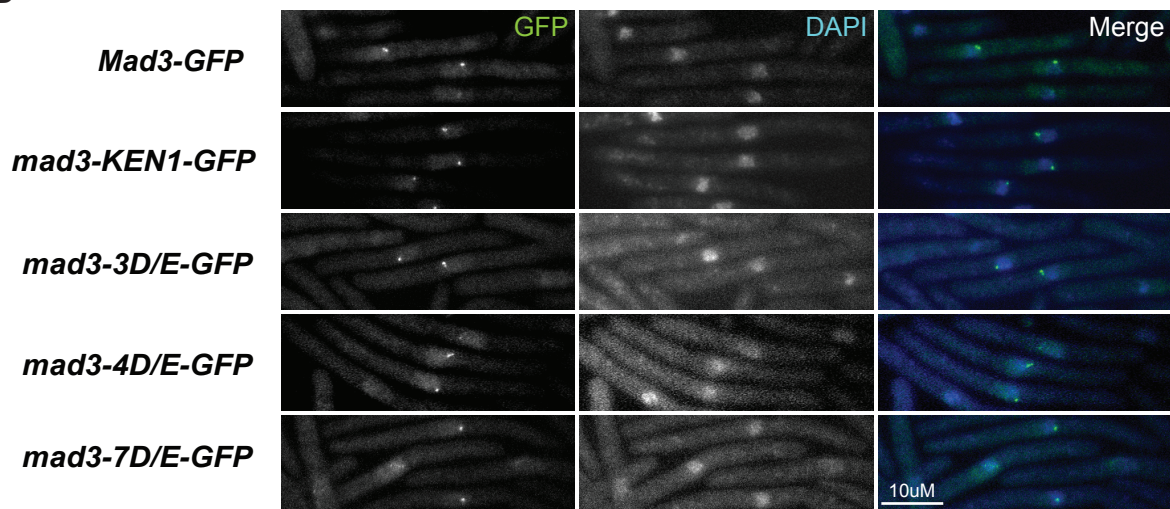

**C**

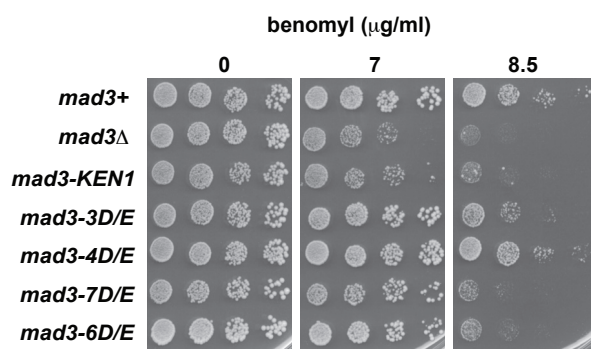

**D**

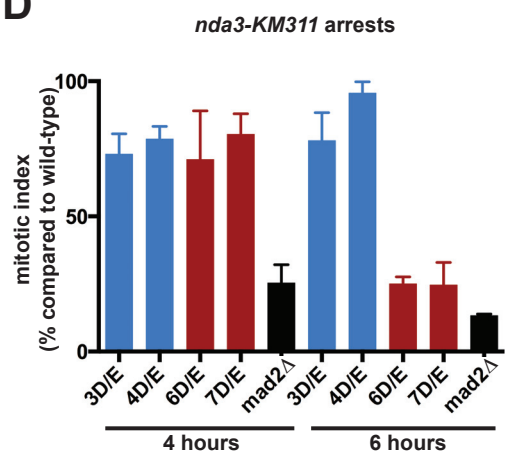

Supplement: S7 Fig — (A) The recombinant Mad3-MBP fusions are all soluble and stable. The gel shown here was stained with coomassie. (B) Mad3 phospho-mimics are targeted to kinetochores. The indicated strains were synchronised in G2 using the cdc25 block, and then released into mitosis before imaging. Scale bar is 10 microns. (C) The mad3-3D/E and 4D/E phospho-mimic alleles are not significantly benomyl sensitive, but the mad3-6D/E and 7D/E alleles are sensitive. Strains were plated on rich (YES) media containing the indicated concentrations of the microtubule disrupting drug benomyl, and grown at 30°C for 3 days. (D) The mad3-3D/E and 4D/E phospho-mimic alleles are checkpoint competent, but the mad3-6D/E and 7D/E alleles are unable to maintain an nda3 arrest. nda3 strains containing the indicated mad3 alleles, or the mad2 deletion as a control, were shifted to 18°C for 4 and 6 hours. Plo1-GFP, which binds SPBs in mitosis, was used to score the mitotic index. The mad3-3D/E and -4D/E alleles arrest well. The mad3-6D/E and -7D/E alleles initially arrest (4 hours) but are unable to maintain the checkpoint arrest and by 6 hours have a significant defect. This indicates that having six or more constitutive phospho-mimic mutations near KEN2 perturbs Mad3p function. (PDF) [file pgen.1005834.s007.pdf]
