# Supplementary material for: Mps1Mph1 Kinase Phosphorylates Mad3 to Inhibit Cdc20Slp1-APC/C and Maintain Spindle Checkpoint Arrests
Source: PLoS Genet. 2016 Feb 16;12(2):e1005834. doi: 10.1371/journal.pgen.1005834 (PMC4755545; doi:10.1371/journal.pgen.1005834)
Supplement: S5 Fig — cdc25 strains indicated were pre-synchronised in G2 by shifting to 36°C for 3.5 hours. They were then released at 25°C and time points taken every 15 minutes. Carbendazim (CBZ) was added after 20 minutes. Cells containing Mad3-GFP were fixed in methanol, stained with calcofluor and then scored for septation, which indicates a failure to maintain spindle checkpoint arrest. The images are from the 60 min time point, and represent the strains used in Figs 4 and 5. The scale bar is 5 microns. (PDF) [file pgen.1005834.s005.pdf]

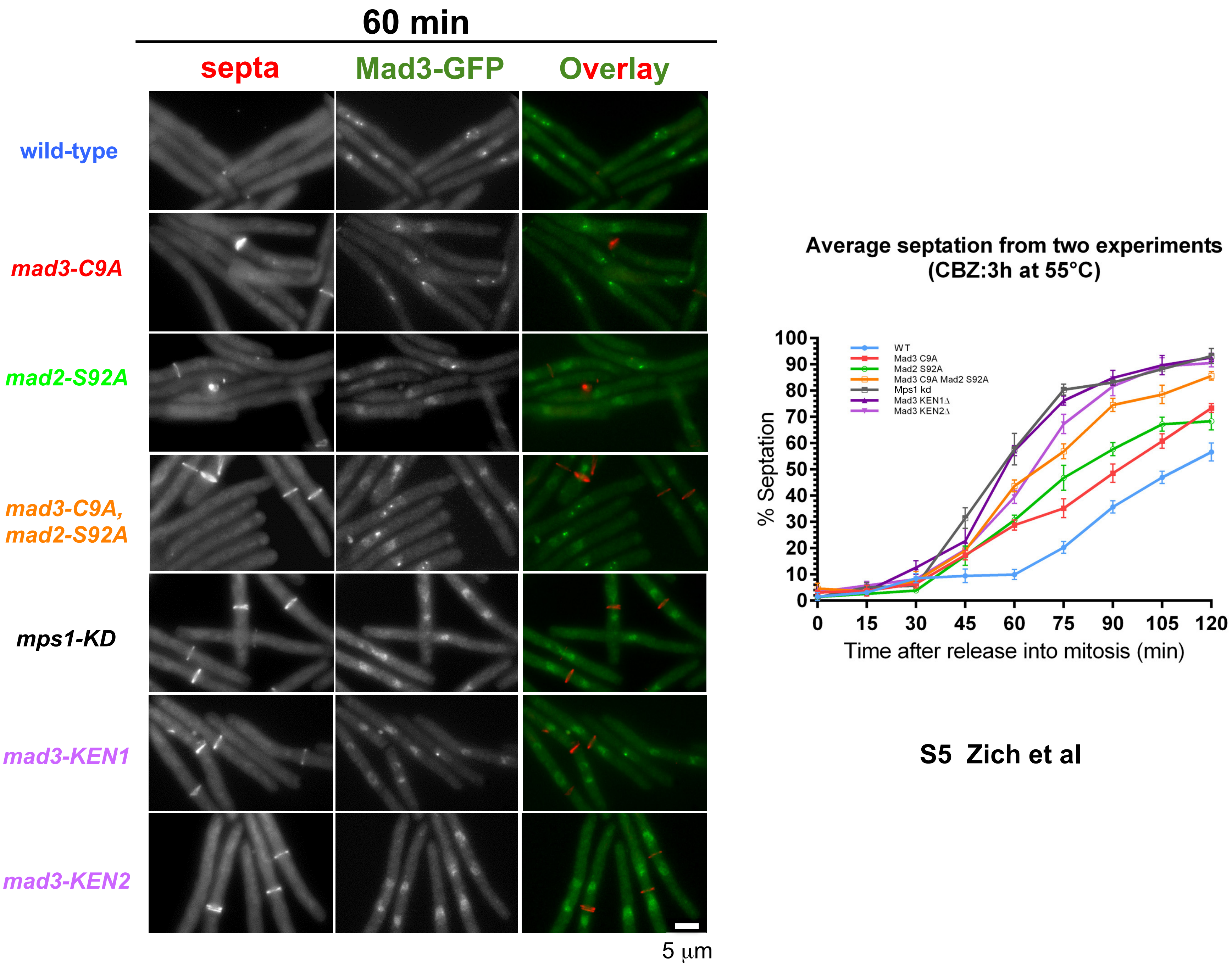

60 min
septa
Mad3-GFP
Overlay
wild-type
mad3-C9A
mad2-S92A
mad3-C9A,
mad2-S92A
mps1-KD
mad3-KEN1
mad3-KEN2
5 μm
Average septation from two experiments
(CBZ:3h at 55°C)
WT
Mad3 C9A
Mad2 S92A
Mad3 C9A Mad2 S92A
Mps1 kd
Mad3 KEN1Δ
Mad3 KEN2Δ
% Septation
100
90
80
70
60
50
40
30
20
10
0
0
15
30
45
60
75
90
105
120
Time after release into mitosis (min)


S5 Zich et al
