# Supplementary material for: Mps1Mph1 Kinase Phosphorylates Mad3 to Inhibit Cdc20Slp1-APC/C and Maintain Spindle Checkpoint Arrests
Source: PLoS Genet. 2016 Feb 16;12(2):e1005834. doi: 10.1371/journal.pgen.1005834 (PMC4755545; doi:10.1371/journal.pgen.1005834)
Supplement: S1 Table — (PDF) [file pgen.1005834.s008.pdf]

S1 Table **Fission yeast strains**

**Figure 1**	SP22	mad3-SZZ::kan^{r} ade6-210 leu1-32 ura4-D18	This lab
	YJZ126	mph1ΔK-GFP mad3-SZZ::kan^{r} ade6-210 leu1-32 ura4-D18	This lab
	YJZ81	leu1^{+}::mph1-kd-SZZ ade6-210 leu1-32 ura4-D18	This lab
	YJZ82	leu1^{+}::mph1-SZZ ade6-210 leu1-32 ura4-D18	This lab
**Figure 2**	MS304	cdc25-22 lid1-TAP::Kan^{r} mad3-GFP::his3^{+} leu1-32 ura4-D18	This lab
	YJZ1086	cdc25-22 lid1-TAP::Kan^{r} leu1^{+}::mad3-C9A-GFP::NAT leu1-32 ura4-D18	This lab
	MS149	cdc25-22 lid1-TAP::Kan^{r} mad3-KEN1-GFP::his3 leu1-32 ura4-D18	This lab
	YJZ795	nuf2-3::ura4^{+} leu1^{+}::mad3 leu1-32 ura4-D18	This lab
	YJZ801	nuf2-3::ura4^{+} leu1^{+}::mad3-N9A leu1-32 ura4-D18	This lab
	YJZ798	nuf2-3::ura4^{+} leu1^{+} ::mad3-C9A leu1-32 ura4-D18	This lab
	YJZ792	nuf2-3::ura4^{+} leu1^{+}::mad3-18A leu1-32 ura4-D18	This lab
	YJZ789	nuf2-3::ura4^{+} leu1^{+}::mad2-dimer leu1-32 ura4-D18	This lab
	MS304	cdc25-22 lid1-TAP::Kan^{r} mad3-GFP::his3^{+} leu1-32 ura4-D18	This lab
	YJZ917	cdc25-22 lid1-TAP::Kan^{r} mad3-GFP::his3^{+} mph1-kd::leu1^{+} leu1-32 ura4-D18	This lab
	YJZ843	cdc25-22 lid1-TAP::Kan^{r} mad3-GFP::his3^{+} leu1^{+}::mad2-S92A leu1-32 ura4-D18	This lab
	YJZ1086	cdc25-22 lid1-TAP::Kan^{r} leu1^{+}::mad3-C9A-GFP::NAT leu1-32 ura4-D18	This lab
	YJZ1082	cdc25-22 lid1-TAP::Kan^{r} leu1^{+}::mad3-C9A-GFP::NAT leu1^{+}::mad2-S92A leu1-32 ura4-D18	This lab
	YJZ914	nda3-KM311 GFP-plo1::ura4 leu1-32 ura4-D18	This lab
	YJZ931	nda3-KM311 GFP-plo1::ura4 leu1^{+}::mad2-S92A leu1-32 ura4-D18	This lab
	YJZ943	nda3-KM311 GFP-plo1::ura4 leu1^{+}::mad3-C9A leu1-32 ura4-D18	This lab
	YJZ1099	nda3-KM311 GFP-plo1::ura4 leu1^{+}::mad2-S92A leu1^{+}::mad3-C9A leu1-32 ura4-D18	This lab
**Figure 3**	OS18	cdc25-22 slp1-FLAG::hph mad3-GFP::his3^{+} leu1-32 ura4-D18	This lab
	KM1218	cdc25-22 slp1-FLAG::hph mad3-GFP::his3^{+} mph1-kd::leu1^{+} leu1-32 ura4-D18	This lab
	KM1253	cdc25-22 slp1-FLAG::hph leu1^{+}::mad3-C9A-GFP::NAT leu1-32 ura4-D18	This lab
	KM1254	cdc25-22 slp1-FLAG::hph leu1^{+}::mad2-S92A-GFP::NAT leu1-32 ura4-D18	This lab
	KM1263	cdc25-22 slp1-FLAG::hph leu1^{+}::mad3-C9A-GFP::NAT leu1^{+}::mad2-S92A leu1-32 ura4-D18	This lab
**Figure 4**	MS304	cdc25-22 lid1-TAP::Kan^{r} mad3-GFP::his3^{+} leu1-32 ura4-D18	This lab
	MS149	cdc25-22 lid1-TAP::Kan^{r} mad3-KEN1-GFP::his3 leu1-32 ura4-D18	This lab
	MS150	cdc25-22 lid1-TAP::Kan^{r} mad3-KEN2-GFP::his3 leu1-32 ura4-D18	This lab
**Figure 5**	YJZ1126	cdc25-22 lid1-TAP::Kan^{r} leu1^{+}::mad3-GFP::NAT leu1-32 ura4-D18	This lab
	YJZ1086	cdc25-22 lid1-TAP::Kan^{r} leu1^{+}::mad3-C9A-GFP::NAT leu1-32 ura4-D18	This lab
	YJZ843	cdc25-22 lid1-TAP::Kan^{r} leu1^{+}::mad3-GFP::his3^{+} leu1^{+}::mad2-S92A leu1-32 ura4-D18	This lab
	YJZ1082	cdc25-22 lid1-TAP::Kan^{r} leu1^{+}::mad3-C9A-GFP::NAT leu1^{+}::mad2-S92A leu1-32 ura4-D18	This lab
	YJZ917	cdc25-22 lid1-TAP::Kan^{r} mad3-GFP::his3^{+} mph1-kd::leu1^{+} leu1-32 ura4-D18	This lab
**S3 Fig**	KP114	ade6-210 leu1-32 ura4-D18	Allshire lab
	KP135	mad3Δ::ura4 leu1-32 ura4-D18	This lab
	YJZ721	leu1^{+}::mad3-C9A leu1-32 ura4-D18	This lab
	YJZ744	leu1^{+}::mad3-N9A leu1-32 ura4-D18	This lab
	YJZ738	leu1^{+}::mad3-18A leu1-32 ura4-D18	This lab
	YJZ1026	leu1^{+}::mad3-C9A+5 leu1-32 ura4-D18	This lab
	MS316	mad3-S289A-GFP::his3^{+} leu1-32 ura4-D18	This lab
	OS18	cdc25-22 slp1-FLAG::hph mad3-GFP::his3^{+} leu1-32 ura4-D18	This lab
	KM1218	cdc25-22 slp1-FLAG::hph mad3-GFP::his3^{+} mph1-kd::leu1^{+} leu1-32 ura4-D18	This lab

	KM1347	*cdc25-22 slp1-FLAG::hph mad3-S289A -GFP::his3*$^{+}$ *leu1-32 ura4-D18*	This lab
**S4 Fig**	MS304	*cdc25-22 lid1-TAP::Kan*^{r} *mad3-GFP::his3*$^{+}$ *leu1-32 ura4-D18*	This lab
	YJZ917	*cdc25-22 lid1-TAP::Kan*^{r} *mad3-GFP::his3*$^{+}$ *mph1-kd::leu1*$^{+}$ *leu1-32 ura4-D18*	This lab
	YJZ843	*cdc25-22 lid1-TAP::Kan*^{r} *mad3-GFP::his3*$^{+}$ *leu1*$^{+}$*::mad2-S92A leu1-32 ura4-D18*	This lab
	YJZ1086	*cdc25-22 lid1-TAP::Kan*^{r} *leu1*$^{+}$*::mad3-C9A-GFP::NAT leu1-32 ura4-D18*	This lab
	YJZ1082	*cdc25-22 lid1-TAP::Kan*^{r} *leu1*$^{+}$*::mad3-C9A-GFP::NAT leu1*$^{+}$*::mad2-S92A leu1-32 ura4-D18*	This lab
**S5 Fig**	MS149	*cdc25-22 lid1-TAP::Kan*^{r} *mad3-KEN1-GFP::his3 leu1-32 ura4-D18*	This lab
	MS150	*cdc25-22 lid1-TAP::Kan*^{r} *mad3-KEN2-GFP::his3 leu1-32 ura4-D18*	This lab
	OS18	*cdc25-22 slp1-FLAG::hph mad3-GFP::his3*$^{+}$ *leu1-32 ura4-D18*	This lab
	KM1218	*cdc25-22 slp1-FLAG::hph mad3-GFP::his3*$^{+}$ *mph1-kd::leu1*$^{+}$ *leu1-32 ura4-D18*	This lab
	KM1253	*cdc25-22 slp1-FLAG::hph leu1*$^{+}$*::mad3-C9A-GFP::NAT leu1-32 ura4-D18*	This lab
	KM1254	*cdc25-22 slp1-FLAG::hph mad3*$^{+}$ *leu1*$^{+}$*::mad2-S92A-GFP::NAT leu1-32 ura4-D18*	This lab
	KM1263	*cdc25-22 slp1-FLAG::hph leu1*$^{+}$*::mad3-C9A-GFP::NAT leu1*$^{+}$*::mad2-S92A leu1-32 ura4-D18*	This lab
**S6 Fig**	OS18	*cdc25-22 slp1-FLAG::hph mad3-GFP::his3*$^{+}$ *leu1-32 ura4-D18*	This lab
	KM1218	*cdc25-22 slp1-FLAG::hph mad3-GFP::his3*$^{+}$ *mph1-kd::leu1*$^{+}$ *leu1-32 ura4-D18*	This lab
	KM1253	*cdc25-22 slp1-FLAG::hph leu1*$^{+}$*::mad3-C9A-GFP::NAT leu1-32 ura4-D18*	This lab
	KM1254	*cdc25-22 slp1-FLAG::hph leu1*$^{+}$*::mad2-S92A-GFP::NAT leu1-32 ura4-D18*	This lab
	KM1263	*cdc25-22 slp1-FLAG::hph leu1*$^{+}$*::mad3-C9A-GFP::NAT leu1*$^{+}$*::mad2-S92A leu1-32 ura4-D18*	This lab
**S7 Fig**	MS304	*cdc25-22 lid1-TAP::Kan*^{r} *mad3-GFP::his3*$^{+}$ *leu1-32 ura4-D18*	This lab
	MS149	*cdc25-22 lid1-TAP::Kan*^{r} *mad3-KEN1-GFP::his3 leu1-32 ura4-D18*	This lab
	YJZ1196	*cdc25-22 lid1-TAP::Kan*^{r} *leu1*$^{+}$*::mad3-3D/E-GFP::NAT leu1-32 ura4-D18*	This lab
	YJZ1194	*cdc25-22 lid1-TAP::Kan*^{r} *leu1*$^{+}$*::mad3-4D/E-GFP::NAT leu1-32 ura4-D18*	This lab
	YJZ1198	*cdc25-22 lid1-TAP::Kan*^{r} *leu1*$^{+}$*::mad3-7D/E-GFP::NAT leu1-32 ura4-D18*	This lab
	KP114	*ade6-210 leu1-32 ura4-D18*	Allshire lab
	KP135	*mad3Δ::ura4 ade6-210 leu1-32 ura4-D18*	This lab
	KP404	*mad3-KEN1-GFP::his3 ade6-210 leu1-32 ura4-D18*	This lab
	YJZ1143	*leu1*$^{+}$*::mad3-3D/E ade6-210 leu1-32 ura4-D18*	This lab
	YJZ1141	*leu1*$^{+}$*::mad3-4D/E ade6-210 leu1-32 ura4-D18*	This lab
	YJZ1135	*leu1*$^{+}$*::mad3-6D/E ade6-210 leu1-32 ura4-D18*	This lab
	YJZ1148	*leu1*$^{+}$*::mad3-7D/E ade6-210 leu1-32 ura4-D18*	This lab
	YJZ1171	*nda3-KM311 plo1-GFP::ura4*$^{+}$ *leu1*$^{+}$*::mad3-3D/E leu1-32 ura4-D18*	This lab
	YJZ1177	*nda3-KM311 plo1-GFP::ura4*$^{+}$ *leu1*$^{+}$*::mad3-4D/E leu1-32 ura4-D18*	This lab
	YJZ1174	*nda3-KM311 plo1-GFP::ura4*$^{+}$ *leu1*$^{+}$*::mad3-6D/E leu1-32 ura4-D18*	This lab
	YJZ1180	*nda3-KM311 plo1-GFP::ura4*$^{+}$ *leu1*$^{+}$*::mad3-7D/E leu1-32 ura4-D18*	This lab
	YJZ227	*nda3-KM311 plo1-GFP::ura4*$^{+}$ *mad2Δ::ura4*$^{+}$ *leu1-32 ura4-D18*	This lab
